# Spatially resolved analysis of TGF/BMP signalling in pancreatic ductal adenocarcinoma by digital pathology identifies patient subgroups with adverse outcome

**DOI:** 10.1186/s12885-025-14751-3

**Published:** 2025-08-18

**Authors:** Konstantin Bräutigam, Philipp Zens, Stefan Reinhard, Jessica L. Rohrbach, Simon J. Leedham, Anna S. Wenning, Beat Gloor, Viktor H. Koelzer, Martin Wartenberg

**Affiliations:** 1 Centre for Evolution and Cancer, Institute of Cancer Research, London, UK; 2 Institute of Tissue Medicine and Pathology, University of Bern, Bern, Switzerland; 3 Graduate School for Health Sciences, University of Bern, Bern, Switzerland; 4 Department of Pathology and Molecular Pathology, University Hospital Zurich, University of Zurich, Zurich, Switzerland; 5 Intestinal Stem Cell Biology Lab, Wellcome Centre for Human Genetics, University of Oxford, Oxford, UK; 6 Translational Gastroenterology Unit, John Radcliffe Hospital, University of Oxford, Oxford National Institute for Health Research Biomedical Research Centre, Oxford, UK; 7 Department of Visceral Surgery and Medicine, Inselspital Bern, Bern University Hospital, University of Bern, Bern, Switzerland; 8 Institute of Medical Genetics and Pathology, University Hospital Basel, University of Basel, Basel, Switzerland

**Keywords:** Transforming growth factors, Bone morphogenetic proteins, Stromal cells, Pancreatic neoplasms, AI (Artificial intelligence), GREM1 protein, Inhibitor of differentiation protein 1 (ID1), Spatial analysis

## Abstract

**Background:**

Transforming Growth Factor (TGF) and Bone Morphogenetic Protein (BMP) signalling critically influence pancreatic ductal adenocarcinoma (PDAC) progression, with TGF-B paradoxically exerting both tumour-promoting and -suppressive effects. Parallel to this observation, the specific context-dependent, spatial dynamics of these pathways and their interaction with the tumour microenvironment (TME) remain poorly understood.

**Methods:**

We performed a spatially resolved analysis of PDAC on a multi-region tissue microarray cohort of 117 curatively resected PDAC specimens consisting of tumour centre (TC), tumour front (TF), and stromal(-predominant) tissue cores each. Protein (ID1, pSMAD2) and mRNA (TGF-A, TGF-B1/2, BMP4, GREM1) expression were assessed in each tissue compartment by immunohistochemistry and in situ hybridization, respectively, quantified by digital image analysis, and correlated with clinicopathologic features.

**Results:**

ID1 was significantly overexpressed in PDAC cells compared to associated stroma (*p* < 0.01), while pSMAD2 was largely absent in PDAC cells, but preserved among associated stroma compartments, particularly in TF cores (*p* = 0.04). Higher stromal GREM1 signal correlated with reduced overall tumoural ID1 protein expression (*p* = 0.02), and TGF-B2^high^/TGF-A^low^ stroma was significantly associated with worse survival (*p* < 0.01). Intratumoural TGF-B2 was inversely correlated with stromal pSMAD2 expression (*p* = 0.03) and was associated with lymph node involvement (*p* = 0.02). FOXP3^+^ regulatory T-cells were significantly reduced in TGF-B2^high^ tumours (*p* = 0.04), while higher tumoural TGF-B1 exhibited a trend towards increased FOXP3^+^ cells (*p* = 0.08).

**Conclusions:**

Our spatial analysis reveals intratumoural heterogeneity of TGF/BMP signalling and its significance for PDAC progression. Notably, stromal TGF-B2 emerges as a prognostic biomarker, while TGF-B1 and ID1 are implicated in adverse clinical and pathologic features. These findings highlight the importance of TGF/BMP signalling niches in the TME with implications for PDAC biology and can inform the development of future therapeutic strategies.

**Supplementary Information:**

The online version contains supplementary material available at 10.1186/s12885-025-14751-3.

## Introduction

Transforming Growth Factor (TGF) and Bone Morphogenetic Protein (BMP) signalling play a crucial role in cancer development and progression [1, 2]. In pancreatic ductal adenocarcinoma (PDAC), one of the most lethal cancer types with increasing incidence [3], TGF-B is known to play a paradoxical role, i.e., it can be tumour-promoting (especially in late-stage, metastatic PDAC) or -suppressive [4, 5]. Promisingly, blocking TGF-B1/B2 enhances sensitivity to combination chemotherapy in PDAC [6], and silencing TGF-B2 expression inhibits tumour proliferation in PDAC cell lines [7] and can boost gemcitabine chemosensitivity [8].

**Fig. 1:**
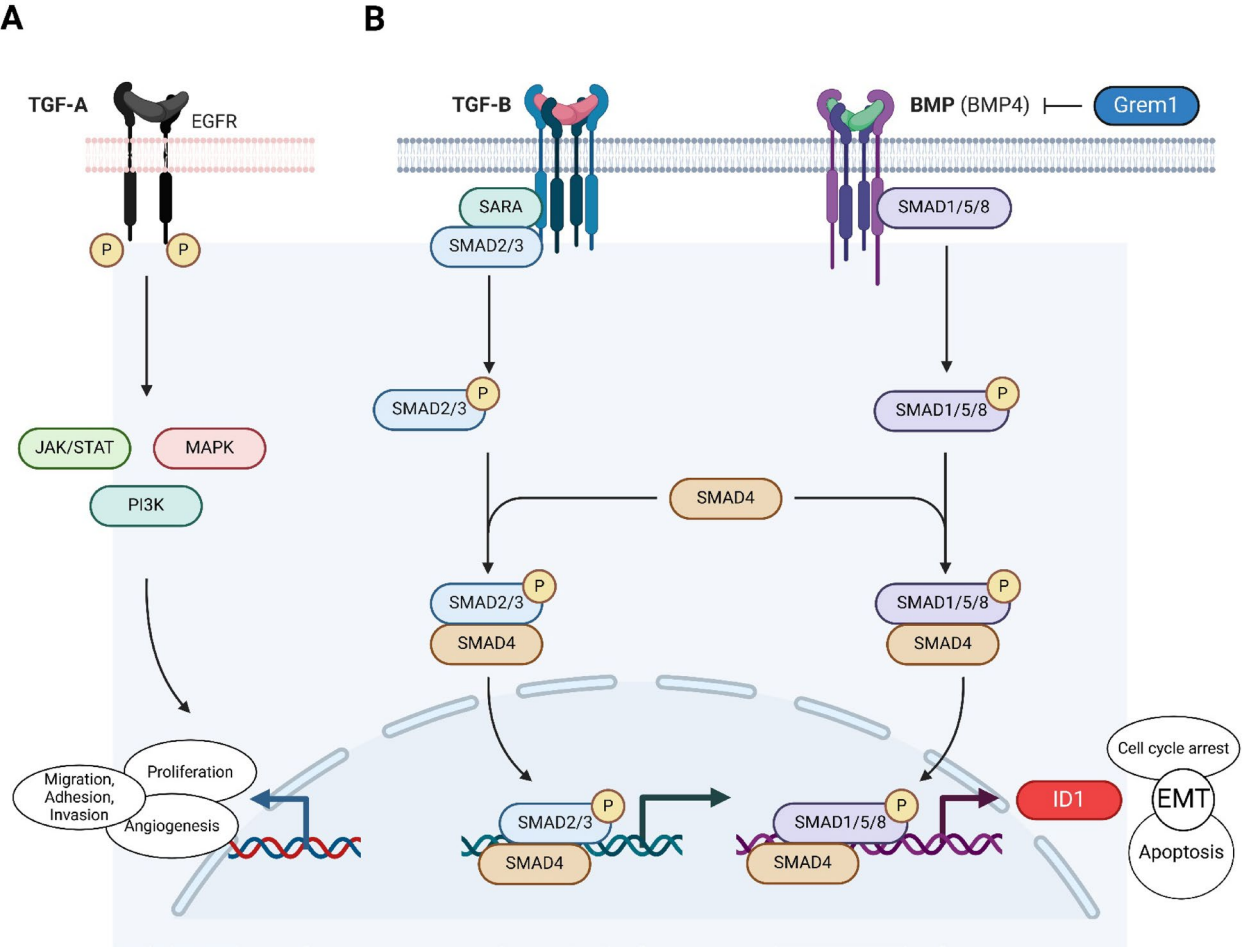
Transforming Growth Factor (TGF)-A, -B and Bone Morphogenetic Protein (BMP) signalling. **A** TGF-A acts as a ligand for the epidermal growth factor receptor (EGFR), leading to receptor dimerization and autophosphorylation, which activates key signalling pathways such as JAK-STAT, MAPK, and PI3K-AKT. This activation promotes essential cellular processes such as (cell) proliferation and angiogenesis. **B** SARA (Smad Anchor for Receptor Activation) facilitates the recruitment of SMAD2/3 to the TGF-B receptor complex. Upon ligand binding, SMAD2/3 becomes phosphorylated (“P”). Once activated, phosphorylated SMAD2/3 dissociates from the receptor and forms a heteromeric complex with SMAD4, which translocates to the nucleus to activate transcriptional programs that regulate processes such as epithelial-mesenchymal transition (EMT), apoptosis, and cell cycle arrest, while also inducing the expression of DNA-binding protein inhibitor (ID1) protein. In BMP signalling, ligands such as BMP4 bind to their respective receptors, leading to the phosphorylation of SMAD1/5/8, which then associates with SMAD4 to form a transcriptionally active complex that also translocates to the nucleus. SMAD4 serves as a common mediator for both the TGF-B and BMP signalling pathways. Gremlin1 (GREM1) functions as a BMP antagonist, binding to BMPs and inhibiting their interaction with BMP receptors, thereby modulating BMP signalling during development and maintenance of tissue homeostasis. Created in *Biorender.com*

TGF-B is a well-known inducer of epithelial-mesenchymal transition (EMT) [4], a process that alters cytoskeletal structure and cellular motility, often enhancing the metastatic potential of cancer cells, and driving a more aggressive phenotype [9, 10] with nuclear abnormalities and chromatin remodelling [11]. High-grade tumour budding, as a morphological “correlate” of EMT [12], has been shown to be a strong predictor of worse outcome in PDAC [13, 14]. Despite the established roles of BMPs and Suppressor of Mothers against Decapentaplegic (SMAD) proteins in regulating EMT and invasiveness, their specific influence on tumour budding in PDAC remains poorly understood [15]. The tumour-promoting effect of TGF-B is pronounced in the context of *SMAD4* loss [4], a gene involved in pancreatogenesis and frequently inactivated in PDAC [16]. SMAD2, a downstream intracellular mediator activated by TGF-B, forms a heterodimeric complex with SMAD4 [17] (Fig. 1). After nuclear translocation, the SMAD2/SMAD4 complex controls gene expression of various processes, such as apoptosis, cell cycle arrest or EMT [18]. TGF-B also has a profound effect on the tumour microenvironment (TME) in PDAC, e.g., by inducing heterogeneity of cancer-associated fibroblasts (CAFs) [19]. TGF-A signalling through the epidermal growth factor receptor (EGFR) has been shown to activate pancreatic stellate cells and may contribute to chronic pancreatitis and pancreatic carcinogenesis [20].

Crosstalk between the TGF-B and BMP pathways exists: Upon binding to BMP receptors, BMPs first activate SMAD1/5/8 and then SMAD4 as the common downstream protein in both pathways (Fig. 1). BMP signalling induces the expression of Inhibitor of DNA binding 1 (ID1), which is known to be upregulated in pancreatitis and PDAC [21, 22] and is believed to have tumour-promoting effects by enabling bypassing of TGF-B signalling [23]. Protein overexpression of ID1 has been described as a negative prognostic predictor associated with higher tumour (T)- and nodal (N)-stage as well as higher microvessel density in PDAC [24]. Interestingly, ID1/ID3 knockdown seems to inhibit PDAC metastasis in human pancreatic cancer cells [25]. Gremlin1 (GREM1) is a BMP antagonist with regulatory function on cellular heterogeneity in PDAC [26]. Its inactivation promotes EMT and lower GREM1 levels are associated with late-stage disease. Recent evidence suggests that GREM1 also influences the TME by driving a fibrogenic activated stroma [27] with higher macrophage counts [28]. An additional microenvironmental factor is TGF-B-induced immune escape, e.g., by recruiting FOXP3^+^ T-cells [29, 30], and thereby turning the tumour into a “cold cancer” with worse outcome. The creation of an immunosuppressive, tumour-permissive (modulatory) niche is supported by the typical context of (excessive) desmoplasia in PDAC, analogous to wound healing [31].

In this work, we spatially resolve and comprehensively quantify key signalling molecules of both, TGF and BMP, pathways in PDAC and the immediate environment of invading cancer cells using digital pathology and a combined protein and mRNA expression profiling approach.

## Materials and methods

### Patient cohort and PDAC tissue microarray (TMA)

Our tissue microarray (TMA) contains 117 cases of curatively resected PDAC treated at the Department of Visceral Surgery of Inselspital Bern, including thirteen with neoadjuvant treatment (cohort details in Table 1, Table S1 and in [32]), and a mean (median; range) follow-up of 794.6 (567; 3 to 3000) days. To account for tumor heterogeneity, each patient case is represented by three tissue cores selected by an expert pathologist (MW) on morphologic grounds from different tumour regions, core diameter 0.6 mm/area 0.28 mm^2^: Tumour front (TF) to account for the infiltrative capacity of the cancer, i.e., the outermost tumour periphery, which is further characterised by histological hallmarks of invasion, e.g. tumour buds and single cell growth; Tumour centre (TC) sampled from the central tumour mass; stroma(-predominant) (in total *n* = 351 tissue cores).

**Table 1:** Clinico-pathological details of the PDAC cohort (*n* = 104) without neoadjuvant treatment (neoadjuvant in Table S1)

Characteristic	*N* = 104^b^
Age (years)	68.6 (SD 9.6)
Gender	
Female	45 / 104 (43.3%)
Male	59 / 104 (56.7%)
Deceased	78 / 103 (75.7%)
NA	1
Overall Survival (days)	801.3 (SD 674.2)
NA	2
Type of resection	
Left resection	11 / 104 (10.6%)
Left resection and adrenalectomy	1 / 104 (1.0%)
Left resection, colectomy and adrenalectomy	1 / 104 (1.0%)
Total pancreatectomy	16 / 104 (15.4%)
Whipple procedure	75 / 104 (72.1%)
Size invasive tumour (mm)	35.3 (SD 11.9, range 12 to 95)
Intrapancreatic location invasive tumour	
Head	81 / 104 (77.9%)
Head and body	1 / 104 (1.0%)
Head, body and tail	2 / 104 (1.9%)
Tail	8 / 104 (7.7%)
Body	7 / 104 (6.7%)
Body and tail	5 / 104 (4.8%)
T-Stage (UICC 8th)	
1c	5 / 104 (4.8%)
2	69 / 104 (66.3%)
3	30 / 104 (28.8%)
N-Stage (UICC 8th)	
0	19 / 104 (18.3%)
1	42 / 104 (40.4%)
2	43 / 104 (41.3%)
M-Stage (UICC 8th)	
0	103 / 104 (99.0%)
1	1 / 104 (1.0%)
Lymphatic vessel infiltration (L1)	82 / 104 (78.8%)
Blood vessel infiltration (V)	
0	12 / 104 (11.5%)
1	89 / 104 (85.6%)
2	3 / 104 (2.9%)
Perineural infiltration (Pn1)	101 / 104 (97.1%)
Resection status (R)	
0	64 / 104 (61.5%)
1	31 / 104 (29.8%)
2	2 / 104 (1.9%)
x	7 / 104 (6.7%)
Grading (G)	
1	12 / 104 (11.5%)
2	51 / 104 (49.0%)
3	40 / 104 (38.5%)
4^a^	1 / 104 (1.0%)
Tumour budding (ITBCC)	
BD0	2 / 104 (1.9%)
BD1	29 / 104 (27.9%)
BD2	31 / 104 (29.8%)
BD3	42 / 104 (40.4%)
Total lymph node (LN) yield	30.0 (12.0)
Positive LN	4.6 (SD 5.3)
Presence of extracapsular extension	46 / 104 (44.2%)

ADM, Acinar-to-Ductal metaplasia; BD, tumour budding; ITBCC, international tumour budding consensus conference; IPMN, intraductal papillary mucinous neoplasm; NA, not available; PanIN, pancreatic intraepithelial neoplasia; SD, standard deviation. UICC, union internationale Contre Le cancer

^a^The grade 4 (G4) tumour represents an undifferentiated carcinoma, anaplastic type.

^b^Mean (SD); n / N (%)

### Immunohistochemistry (IHC)

Primary antibodies were incubated for 30 min at room temperature and used as follows: Anti-Id1 (ab203202, rabbit polyclonal, Abcam, Cambridge, United Kingdom, dilution: 1:200, retrieval: Citrate buffer pH 6, 20 min); CD8 (M710301, mouse monoclonal, clone C8/144B, DAKO/Agilent Technologies, CA, United States, dilution 1:200, retrieval: TrisEDTA, 20 min); CD68 (M087601, clone PG-M1, monoclonal mouse, DAKO/Agilent Technologies, dilution: 1:200,Tris EDTA, 30 min); CD163 (NCL-CD163, clone 10D6, mouse monoclonal, Leica Biosystems, Newcastle upon Tyne, United Kingdom, dilution: 1:400, retrieval: Tris EDTA, 20 min); FOXP3 (14-477-82, mouse monoclonal, clone 236 A/E7, Invitrogen, MA, United States, dilution: 1:50, retrieval: Citrate buffer pH 6, 30 min); pSMAD2 (3108 S, rabbit monoclonal, Cell Signaling Technology, London, United Kingdom, dilution: 1:200, retrieval: Citrate buffer pH 6, 20 min). Antibody detection was performed with the BOND Polymer Refine DAB kit (Leica Biosystems) using 3,3-diaminobenzidine as a brown chromogen. The samples were counterstained with hematoxylin. TMA slides were scanned on a 3DHISTECH Pannoramic 250 Flash II scanner (3DHISTECH, Budapest, Hungary). Protein expression was rigorously validated by three board-certified pathologists (KB, VHK and MW) and by including appropriate on-slide controls.

### Messenger RNA (mRNA) in-situ hybridisation (ISH)

mRNA transcripts of TGF-A (reg 58, reference 313138 Hs-TGFA), TGF-B1 (reg 52, reference 400888 Hs-TGFB1), TGF-B2 (reg 54, reference 489248 Hs-TGFB2), BMP ligand BMP4 (reg 56, reference 454308 Hs-BMP4), and BMP antagonist GREM1 (reg 46, reference 312831 Hs-GREM1) were visualized by applying RNAscope (2.5 LS DUPLEX, Reagent Kit-BROWN) chromogenic probes (ACD BioTechne, Newark, CA, USA) on the TMA sections described above using TrisEDTA epitope retrieval buffer (95°, 20 min) and ACD enzyme for 15 min, together with a negative control probe (2.5 LS Duplex “DapB”).

### Digital image analysis (DIA)

**Fig. 2:**
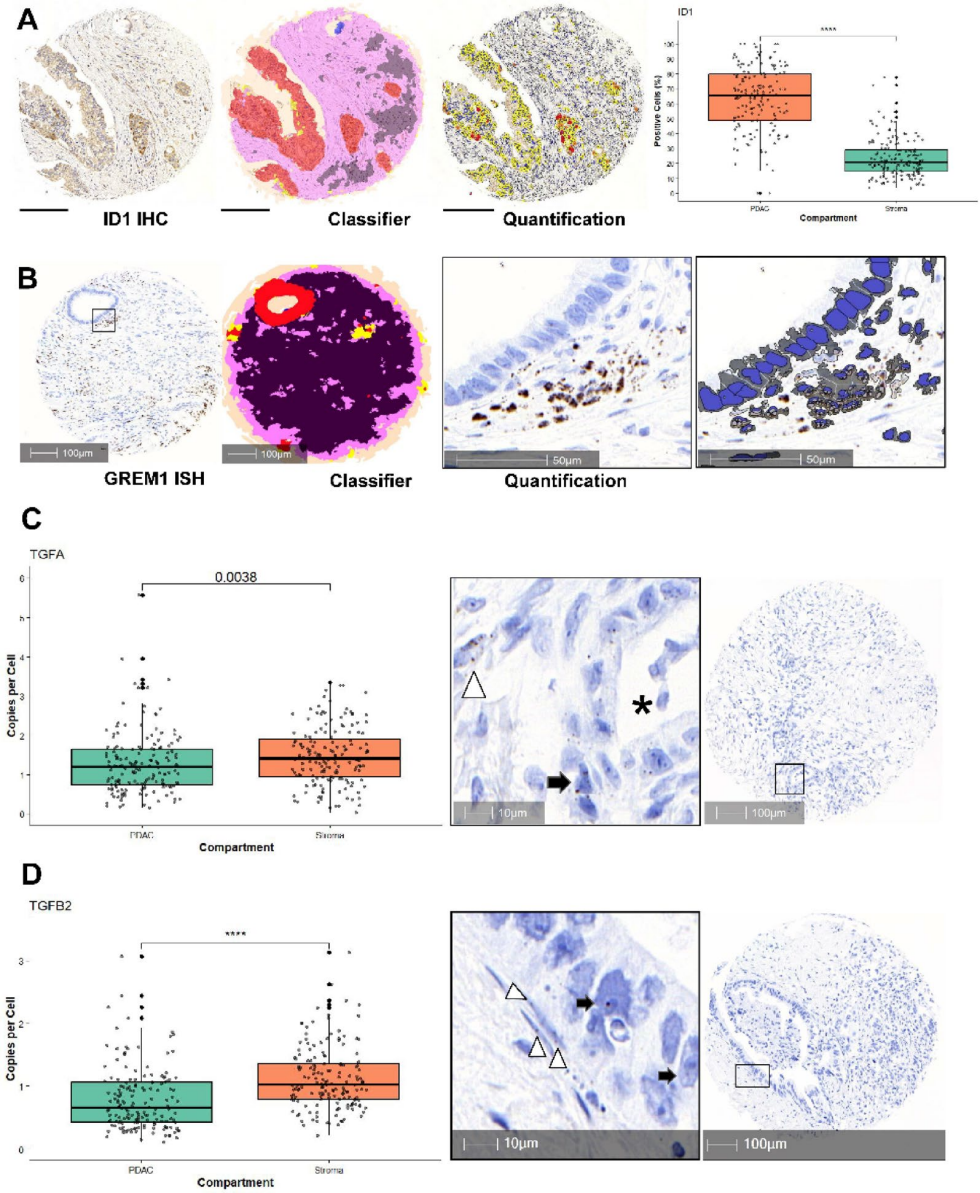
Digital Image Analysis (DIA) and examples of expression of key markers across spatial compartments. **A**,** B** Immunohistochemistry (IHC): High ID1 protein expression in PDAC parenchyma. mRNA in-situ hybridisation (ISH): Strong stromal/juxtatumoural GREM1 signalling with high mRNA transcript counts (inset). Extended in Figure S1. *Black* and *purple*: stroma, *Blue*: artifact, *Red*: PDAC parenchyma, *Yellow*: necrosis. *Scale bar* in panel A: 200 μm. **C**, left TGF-A signalling in PDAC epithelium (arrow, asterisk) and stroma (arrowhead) with significantly higher mRNA copies per cell in tumour stroma. **C**, right, example of TGF-A mRNA ISH in the PDAC TME **D**, left Significantly stronger TGF-B2 signalling in tumour stroma (arrowheads) compared to PDAC epithelium; **D**, right example of TGF-B2 mRNA ISH in the PDAC TME (arrows). *TME*: Tumour Microenvironment

DIA was performed using HALO AI™ (Indica Labs, NM, USA). The digitized TMA slides were de-arrayed into individual spot images of each tissue sample, which were then linked to the corresponding clinical annotations. After the first visual review, seven tissue cores with insufficient tissue and/or artifacts were excluded from further analysis. To localize and quantify tumour and stromal tissue, a deep neural network algorithm was trained based on a pre-trained DenseNet network (Fig. 2A and B). Graphical overlays were generated for each tissue class and the classification accuracy was visually verified. The total area of each tissue class was quantified in square millimetres (mm^2^). Nuclear segmentation and staining quantification were performed in both the tumour and stromal compartments. For nuclear segmentation, the HALO™ AI pre-trained convolutional neural network was used and fine-tuned with application-specific training examples. Pixels were classified as positive if the staining intensity exceeded the internal controls, as validated by pathologist review (KB, VHK). The total area of vital tissue in the tumour and stromal compartments, the number of ID1-positive and pSMAD2-positive cells (*Cytonuclear* v2.0.9 module), and mRNA transcripts of TGFA, TGFB1, TGFB2, BMP4 and GREM1 (*ISH IHC* v3.2.5 module) were recorded for analysis with clinicopathologic features. Marker quantification subproperties such as cytoplasmic radius, contrast thresholds, optical density, segmentation aggressiveness, and nuclear staining intensity were optimized based on expert pathologist review. Nuclear roundness (from 0 to 1, with 1 being perfectly round) and cytoplasmic radius were measured by HALO™ Image Analysis Software.

Algorithmic outputs for ISH and IHC—marker localisation and expression intensity—were reviewed by three expert pathologists (KB, VHK, MW). Results were visualized as human-interpretable tissue mark-ups, which were independently reviewed for accuracy and plausibility. Ambiguous cases were resolved by consensus discussion. Final validation included consistency checks against known biological patterns, expected staining behaviour, cellular localisation, and published data. The accuracy of the tumour–stroma segmentation was formally validated using a subset of tissue cores (Table S7).

### Tumour budding and immune cell quantification

The grade of tumour budding was assigned by consensus (BD0 to BD3) [14] for each tumour using representative hematoxylin and eosin (H&E) slides. In addition, tumour budding was individually counted at the tissue core level for each individual marker by an expert pathologist (MW) in a blinded, independent and randomized manner using the browser-based online TMA analysis tool “Scorenado” [33]. CD8^+^ T-cells, FOXP3^+^ T-regulatory cells and macrophages (CD68^+^ and CD163^+^) have been binarily classified into “low” and “high” per tissue core (MW).

### Validation using The Cancer Genome Atlas (TCGA)

The findings were independently validated using open-source, publicly available data from The Cancer Genome Atlas (TCGA). mRNA data (pancreatic adenocarcinoma (PAAD), *n* = 177) were accessed and plots were generated using the online tool “UALCAN: A portal for facilitating tumor subgroup gene expression and survival analyses” (https://ualcan.path.uab.edu/cgi-bin/ualcan-res.pl*)* [34].

### Statistical analysis

For statistical analyses, only tissue cores with at least 25 tumour and stromal cells (corresponding to approximately the 25th percentile of epithelial cell counts in the raw data output) were used (thereby losing all tissue cores from a total of five patients). In the non-neoadjuvant cases (*n* = 104), the PDAC cell count ranged from 25 to 1605 cells per tissue core (median: 218; mean: 305; interquartile range: 290), while the stromal cell count ranged from 42 to 1862 cells per tissue core (median: 638; mean: 712; interquartile range: 430). Unless stated otherwise, cases involving neoadjuvant therapy were excluded from downstream statistics. Statistical analyses were performed using the R statistical computing environment version 4.4.1 (RStudio version 2024.04.2). All *p*-values were calculated for two-tailed tests with significance set at *p* < 0.05. Statistical analyses included Wilcoxon signed-rank tests for pairwise comparisons, Spearman’s rank correlation coefficient to assess associations, Kaplan-Meier survival analysis with log-rank tests for time-to-event data, and chi-squared tests to assess relationships between categorical variables.

## Results

### ID1 is highly expressed, while pSMAD2 protein mostly absent in PDAC parenchyma

ID1 protein was highly expressed among epithelial PDAC cells compared to the surrounding stroma (*p* < 0.01, non-neoadjuvant cases) in both TC and TF (Figs. 2A, S1 and S8A, Table 2). Mean ID1 protein expression was even more pronounced in the neoadjuvant-treated cases (both TC and TF, Table S2). pSMAD2 protein expression was mostly absent in PDAC parenchyma, aligning well with TCGA mRNA data (reduced transcript counts compared to normal parenchyma, Figure S8B), with significantly higher preservation in the stroma (*p* < 0.01, non-neoadjuvant cases) (Figure S2A, Table 2 and S2). Mean pSMAD2 expression in PDAC was significantly lower in the TC than in TF (*p* = 0.02) while mean stromal pSMAD2 protein expression was significantly higher in the TF (*p* = 0.04) (non-neoadjuvant cases, Figure S2B). Both PDAC parenchyma and stroma were mostly BMP4^low^ (non-neoadjuvant cases, mean 0.25 mRNA transcripts per tumour cell) with a few parenchymal BMP4^high^ cases (maximum of 6.3 transcripts/tumour cell (TF) in a ATM-mutant pT3 pN1 duct adenocarcinoma [35, 36] with adjuvant FOLFIRINOX [37] and > 5 years overall survival; hyperactive BMP4 signalling described in PDAC with ATM loss [38]; respective tissue core in Figure S1) (Table 2).

**Table 2:** Expression of TGF- and BMP-signalling molecules in PDAC and associated stroma, each for tumour centre (TC) and tumour front (TF) (non-neoadjuvant only, *n* = 82 analysed, neoadjuvant in Table S2). Protein expression (ID1, pSMAD2 immunohistochemistry) in percent (%), mRNA transcripts (in-situ hybridisation) per PDAC cell and stromal cell respectively (including only tissue cores with at least 25 stromal and at least 25 cancer epithelial cells)

Region	PDAC_ID1	Stroma_ID1	PDAC_pSMAD2	Stroma_pSMAD2
TC	63.8% (0–100, SD = 22.7%)	22.7% (3.7–77.7, SD = 13.1%)	16.3% (0–100, SD = 21.3%)	15.6% (0.7–87.8, SD = 18.9%)
TF	60.5% (0–100, SD = 24.4%)	23.9% (3.1–72.6, SD = 12.9%)	16.5% (0–100, SD = 26.4%)	23.7% (0–96.7, SD = 25.1%)

**Region**	**PDAC_GREM1**	**Stroma_GREM1**	**PDAC_TGFA**	**Stroma_TGFA**	**PDAC_TGFB1**	**Stroma_TGFB1**	**PDAC_TGFB2**	**Stroma_TGFB2**	**PDAC_BMP4**	**Stroma_BMP4**
TC	0.7 (0-5.5, SD = 1.1)	0.9 (0-6.4, SD = 1.2)	1.3 (0.2-3.4, SD = 0.7)	1.5 (0-3.2, SD = 0.7)	1.8 (0.1-8.5, SD = 1.6)	0.9 (0-2.4, SD = 0.6)	0.8 (0.1-2.3, SD = 0.4)	1.2 (0.4-3.1, SD = 0.5)	0.4 (0-2.4, SD = 0.4)	0.3 (0-1.3, SD = 0.2)
TF	0.6 (0-3.9, SD = 0.7)	1.1 (0-10.9, SD = 1.6)	1.3 (0.2-5.6, SD = 0.9)	1.5 (0.2-3.3, SD = 0.8)	1.8 (0.2-6.1, SD = 1.2)	0.9 (0-2.2, SD = 0.5)	0.8 (0.1-3.1, SD = 0.5)	1.1 (0.2-2.3, SD = 0.4)	0.5 (0-6.3, SD = 0.9)	0.4 (0-1.1, SD = 0.3)

SD, standard deviation

### Strong GREM1 signalling in tumour-associated stroma and robust TGF signalling across compartments

**Fig. 3:**
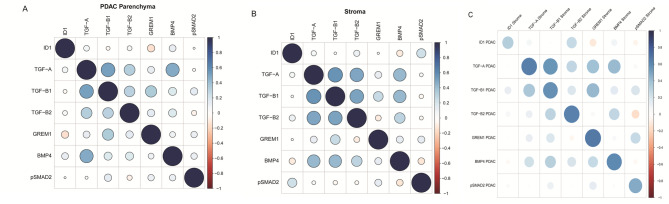
Inter- and intra-compartment correlation. Marker expression (neoadjuvant cases excluded) in PDAC parenchyma (**A**), juxtatumoural stroma (**B**) and tumour parenchyma versus stroma (**C**). Scale indicates Spearman-Rho correlation coefficient and direction of association

GREM1, TGF-A and TGF-B2 mRNA counts per cell were significantly higher in the stromal compartment compared to the PDAC cells (non-neoadjuvant only, Figs. 2B–D; Table 2). On the contrary, TGF-B1 mRNA transcript counts were significantly higher in the PDAC compartment (*p* < 0.01). There were no significant differences between TC and TF in transcript counts for the three investigated TGF ligands, BMP4 and GREM1. Both among the tumoural and stromal compartment, transcript counts of TGF-A, TGF-B1 and -B2 showed strong positive correlations (Fig. 3A and B, Table S3). In PDAC parenchyma, higher GREM1 transcript counts were negatively correlated with ID1 protein expression (rho= − 0.146, *p* = 0.03), consistent with biological priors. When comparing tumoural and stromal metrics, higher stromal GREM1 transcript numbers correlated with higher stromal TGF-A and -B1 transcript counts (rho = 0.40, *p* < 0.01; rho = 0.42, *p* < 0.01, Fig. 3C, Table S3). Higher tumoural BMP4 correlated with stromal TGF-A transcripts (rho = 0.42, *p* < 0.01).

### Low GREM1 signalling and high ID1 expression define an aggressive PDAC phenotype

**Fig. 4:**
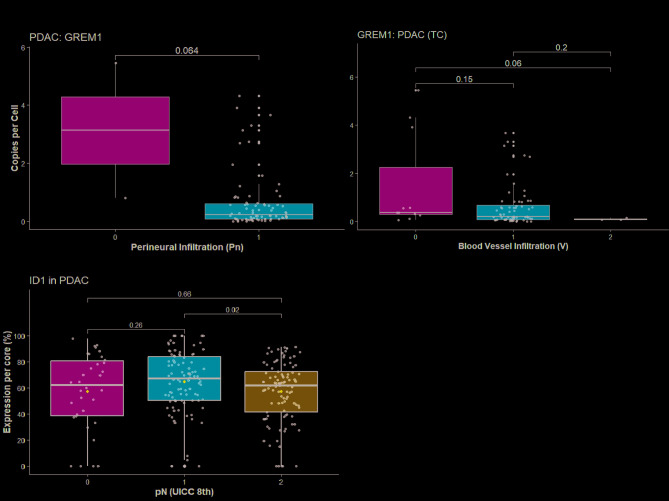
Higher tumour aggression in ID1^high^, TGF-B2^high^ and GREM1^low^ PDAC. **A** Less perineural invasion and (**B**) less (micro- (V1) and macroscopic (V2)) blood vessel infiltration in GREM1^low^ PDAC (TC). **C** Higher ID1 protein expression (in %) in lymph-node positive PDAC (A–C: neoadjuvant cases excluded)

While GREM1^low^ PDACs showed a trend towards perineural invasion (TC: *p* = 0.06, Fig. 4A) and blood vessel infiltration (V0 vs. V2: *p* = 0.06, Fig. 4B), expression of any of the signalling molecules in PDAC (TC and TF) was not significantly associated with the presence of extracapsular extension of lymph node (LN) metastases, lymphovascular (“L1”) or perineural invasion (“Pn1”), tumour stage or tumour grade.

Tumour size correlated weakly with ID1 expression in the TF (*p* = 0.03, R^2^ = 0.06; Figure S3A), but not in the TC (*p* = 0.14). N2-stage PDAC showed lower tumoural ID1 expression than N1-stage (*p* = 0.02; Fig. 4C, N0 vs. N1/N2: *p* = 0.73, TCGA: Figure S8C). Other molecules exhibited no significant associations with tumour size, number of positive LN (Figure S3C and S3D) or N-stage. Lower average GREM1 transcript counts per stromal (TC: *p* < 0.01; TF: *p* = 0.07, t-test) and tumoural cell (TC: *p* < 0.01; TF: ns, t-test) in the TC spots, significantly associated with presence of metachronous distant metastasis in follow-up. TGF-B2 transcript counts per cell in PDAC parenchyma correlated significantly with the absolute number of positive lymph nodes (TF, *p* = 0.02, Figure S3B). TCGA data support this finding (non-significant trend, Figure S8D) and reveal higher tumoral transcript counts in N1-stage PDAC.

TGF-A^low^ (*p* = 0.06, Wilcoxon rank sum; *p* = 0.09, Fisher test) and -B1^low^ stroma (*p* = 0.08, Wilcoxon rank sum; *p* = 0.07, Fisher test) showed a statistically non-significant trend towards higher tumour budding (BD2 and BD3, TC, Figure S4). When quantifying tumour buds per individual tissue core, we found no significant differences in tumour bud counts for any of the signalling molecules studied (Table S4). This held true whether we compared the average marker expression in PDAC versus stroma or between TC and TF per tissue core.

### Heterogeneity of immune infiltrate and sparsity of FOXP3^+^ T-regulatory cells in TGF-B2^high^ PDAC

Immune cell counts were heterogeneously distributed among the different tumour regions. CD3 had the greatest heterogeneity (43.3% of patients had consistent expression patterns (low, high) across tissue cores) in all three tumour regions (TC, TF, stromal-predominant tissue cores), while CD20 had the least (85.5); this pattern persisted when considering only TC and TF (CD3: 63.5%, CD20: 88.5%) (Table S5).

TGF-B is known for its direct suppressive effect on T-cell function and induction of T-regulatory cells [30, 39] and the TF (invasive margin) is known as a site of pronounced immune density in PDAC [40, 41]. In our cohort, we observe a statistically non-significant trend towards higher FOXP3 counts in the TF of TGF-B1^high^ tumours (*p* = 0.08, Figure S5A; TC: *p* = 0.29). Stromal TGF-B1 copies did not associate with FOXP3 levels (TC: *p* = 1; TF: *p* = 0.64). Higher stromal and intratumoural TGF-B2 transcripts, significantly associated with lower FOXP3 counts in the TC (*p* = 0.04 and *p* = 0.05 respectively, Figure S5A). ID1 has been shown to suppress CD8^+^ T-cell infiltration [42]. Here, CD8^high^ tissue cores were not associated with ID1 expression levels (TC: *p* = 0.35, TF: *p* = 0.17, Figure S5B). No significant associations between TGF-B1 or TGF-B2 (in either compartment) and CD8^+^ T-cell infiltration were detected (TC/TF: all non-significant). While BMP4 has been shown to induce M2-polarization of macrophages in bladder cancer [43], we did not observe significant differences in CD68^+^ or CD163^+^ macrophage levels between BMP4^low^ and -^high^ PDAC (TC: *p* = 0.85; TF: *p* = 0.52). Also, GREM1 transcripts have been described to be associated with increased M1- and M2-macrophages in PDAC [28, 44]. Binary (by mean) stratification of BMP4 (CD68, TC: *p* = 0.24; TF *p* = 0.71; CD163, TC: *p* = 0.41; TF: *p* = 0.71) or GREM1 (CD68, TC: *p* = 0.17; TF: *p* = 0.35; CD163, TC: *p* = 0.31; TF: *p* = 0.56) mRNA transcript counts did not show significant correlations with macrophage levels (Figure S5C and -D).

### Stromal TGF-A and -B2 linked to worse PDAC survival

**Fig. 5:**
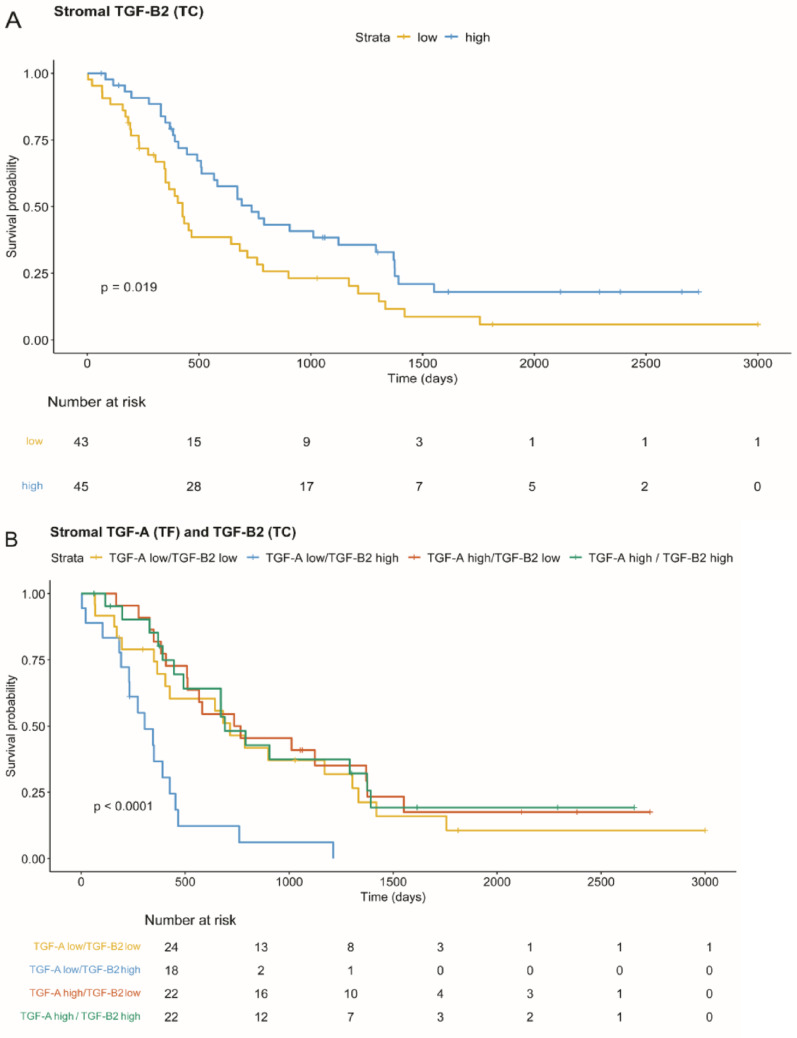
Stromal TGF-A and -B2 define subgroups of worse survival in PDAC. **A** TGF-B2^low^ (TC) stroma and (**B**) TGF-A^low^(TF)-TGF-B2^high^ (TC) stroma significantly associated with worse survival (low vs. high stratified by mean, neoadjuvant cases excluded, other outcome analyses in Figure S6B and C)

Lower number of stromal TGF-B2 copies in the TC were significantly associated with worse survival (*p* = 0.02, neoadjuvant cases excluded, Fig. 5A, multivariate Cox model in Figure S6A). A higher number of stromal TGF-A in the TF showed a statistically non-significant trend towards worse survival (*p* = 0.069). Interestingly, when both stromal TGF-A and -B2 were combined, the poorest overall survival was seen in the TGF-A^low^(TF)-TGFB2^high^(TC) stroma group (*p* < 0.01, Fig. 5B), highlighting the complex interaction between these molecules and their non-linear relationship. Comparison of stromal TGF-A and -B2 groups within the same tumour region did not show statistical significance (TC/TC: *p* = 0.06; TF/TF: *p* = 0.21). Binary survival analysis (low versus high) of bulk mRNA TCGA data did not reveal any significant differences in outcomes for the investigated TGFs (Figure S8E). Neither TGF-B1, BMP4 and GREM1 transcript levels nor ID1 and pSMAD2 protein expression were significantly associated with survival in either tumour or stromal compartments (Table S5).

### “Mesenchymal geometry” and stromal proportion does not correlate with PDAC outcome

To investigate EMT and its impact on tumour aggressiveness in PDAC, we examined nuclear roundness as a morphological EMT surrogate, i.e., more spindle-shaped, less round morphology in the mesenchymal cell spectrum. Nuclear circularity is decreased during EMT induction [45] and, for instance, in breast cancer a “rounded to deformed morphology” has been described after EMT [46]. Here, the automatically computed roundness of PDAC nuclei was between 0.61 and 0.80 (mean of 0.73 among all tissue cores, neoadjuvant cases excluded). There was no significant correlation of nuclear roundness with N-stage in this cohort (*n* = 102, non-neoadjuvant, TC). Further, there was no significant difference in survival comparing less round to rounder PDAC nuclei (*p* = 0.83, TC) (Figure S6**C**). TGF-B is a known (sometimes SMAD-dependent) inducer of tissue fibrosis [47]. In PDAC, the typically dense desmoplastic stroma is traditionally considered a negative prognostic factor and is thought to form a tumour protective “niche” and physical barrier that can reduce drug penetration and prevent immune infiltration. Here, the amount of stroma was not associated with worse survival (neoadjuvant patients excluded, stratified by the mean stromal percentage per tissue core, TF: *p* = 0.23; TC: *p* = 0.32, Figure S6B).

## Discussion

The results of this study on TGF- and BMP-signalling in PDAC align well with the known microenvironmental [48, 49], transcriptomic [50] and cellular heterogeneity of PDAC across space, time and anatomical regions [40, 51, 52]. Recent evidence suggests a site-specific TME [53] with intratumoural heterogeneity on single-cell level [54] involving microenvironmental transitions in metastatic progression of PDAC [52]. In our spatially-resolved analysis investigating multiple tissue cores per PDAC case from different morphological tumour regions to account for tissue heterogeneity, we reveal differences in TGF/BMP signalling and, consistent with previous literature [55–57], in immune cell distribution across tumour regions. A chronic inflammatory state during manifestation of PDAC paralleled by dysregulation of the associated microenvironment might provide a cancer-promoting milieu and allow tumour cells to exploit physiological functional niches during tumour progression (“niche hijacking”) [58, 59].

In mice, SMAD2 and − 3 have been revealed to be crucial molecules that trigger a TGF-B-induced regulatory T-cell response [60]. We observe contradictory results in TGF-B signalling: While TGF-B2^high^ PDAC significantly associated with less FOXP3^+^ T-regulatory cell infiltration in the TC, TGF-B1^high^ PDAC showed the opposite trend. Higher FOXP3^+^ counts in the proximity of TGF-B1^high^ PDAC align well with EMT [61], however, tumoural TGF-B1 mRNA counts themselves were not associated with adverse survival. Notably, here, TGF-B transcript counts were not associated with CD8^+^ T-cell levels. In combined analysis of the tumour regions, TGF-A^low^ (TF)/TGFB2^high^ (TC) stroma was associated with worse overall survival. Interestingly, in the TCGA data, there were no significant outcome differences in compartment-agnostic tumoral TGF mRNA transcripts (high versus -low, *n* = 177), further highlighting the pivotal relevance of spatially resolved expression analysis. Recent data suggests negative LN to be a predictor of chemotherapy (gemcitabine plus capecitabine) efficacy [62]. Here, in the TF, a cancer compartment associated with infiltrative properties [63], elevated tumoural TGF-B2 transcripts correlated with a higher number of lymph node metastases, in line with early reports on the role of TGF-B isoforms on PDAC progression [64] and bulk mRNA TCGA data. The TF has been characterised as a site of elevated immune interaction and immune cell density [41, 65], with upregulation of inflammatory pathways [40, 66] and pronounced tumour aggressiveness, for example in the form of tumour budding [67]. Our findings regarding ID1 expression and TGF-B2 transcript counts in the tumour further emphasise its aggressive biology. Although our regression analyses revealed few significant associations (with outliers largely determining the significance of factors such as lymph node metastases and tumour size), we believe that examining larger cohorts would reinforce these findings. For example, neither tumoural nor stromal BMP4 showed any trend in association with the number of lymph node metastases or tumour size. This further highlights the complexity of pathway activity and the importance of possible downstream dysregulation. Higher TGF-B2 mRNA levels have been reported to be associated with worse overall survival when present in a macrophage-poor TME [68]. It appears that TGF-B2^high^ PDAC and TGF-B2^low^ stroma define an aggressive, pro-metastatic PDAC. The reported effect of TGF-B2 receptor overexpression on survival in PDAC has been variable [69, 70]. In mice, TGF-B2 receptor neutralization resulted in increased differentiation and decreased metastasis of PDAC [71]. This suggests that biological behaviour is strongly dictated by regional compartments/functional neighbourhoods rather than mere pathway activation.

mRNA transcript counts of all TGF-ligands strongly correlated among tumour and stroma, possibly underscoring excessive fibrogenic signalling in PDAC (aberrant “wound healing” [72]). Interestingly, GREM1, TGF-A and TGF-B2 transcripts were significantly enriched in the juxtatumoural stroma as compared to PDAC parenchyma. The strong stromal predilection of GREM1 is in line with previously reported high GREM1-levels in (cancer-associated) pancreatic fibroblasts [28, 44]. Corroborating previous results in SMAD4 [70], SMAD2 protein expression levels were not associated with worse survival. Consistent with proficient downstream BMP-signalling, GREM1^high^ PDAC showed less ID1 expression. As reported [24], we could confirm robust ID1 protein expression in PDAC, regardless of tumour region and in line with TCGA data. In our cohort, ID1^high^ tumours were significantly larger, but there was no further significant association with conventional histopathologic risk predictors or with overall survival. Ultimately, the BMP pathway also leads to cell cycle arrest. ID1 is a downstream protein of this pathway and is, herein, associated with larger tumour size. Consequently, aberrant, context-dependent pathway signalling that bypasses physiological activity (e.g. via upstream molecules) is likely to occur in PDAC. Paradoxically, tumoural ID1 expression was significantly lower in N2- than N1-stage PDAC. ID1 has been shown to exert an immunosuppressive effect by promoting myeloid-derived suppressor cell expansion and downregulating CD8^+^ T-cells [42], while the density of CD8^+^ T-cells in the TC has been demonstrated to have prognostic validity in PDAC [73]. In our cohort, a high CD8^+^ T-cell infiltrate did not correlate with a higher percentage of tumoural or stromal ID1 expression. Of note, this was independent of the tumour region (TC or TF), despite previous evidence suggesting that there is less CD8 infiltration and more immunosuppression in the TC [41, 65]. GREM1 protein expression has been described to coincide with M1/M2 macrophage enrichment in PDAC [28, 44], a finding we cannot reproduce in our binary stratification using mRNA transcript counts and CD68/CD163 immunohistochemistry. Despite the “different faces” of GREM1 function [74], loss of GREM1 promotes metastasis [26] and a fibrogenic stromal microenvironment [28]. In this work, lower stromal and tumoural GREM1 transcript numbers were significantly associated with distant tumour recurrence, supporting the role of GREM1 loss for metastatic seeding.

Interestingly, tumour budding counts did not differ significantly between tumour regions or between expression levels for any of the molecules analysed. TGF-B secretion by stromal cells enhances metastatic capacity in colorectal cancer [75], while in PDAC, it induces a myofibroblastic phenotype of cancer-associated fibroblasts via EGFR/ERBB2 signalling, promoting metastasis [76]. Targeting TGF signalling in PDAC has already shown promising results in combination therapy [6, 8]. Here, we observed trends toward higher tumour budding in TGF-A^low^ and -B1^low^ stroma. We found no distant tumour recurrence in TGF-B1^high^ or -B2^high^ PDAC. The TGF-B “paradox” [77, 78], whereby TGF-B exhibits tumour-suppressive effects in the early stages of PDAC but tumour-promoting effects in the late stages, remains to be deciphered. In our cohort, we observed no significant differences in TGF transcript counts according to T-stage. However, the association of TGF-B2^high^ stroma with a higher number of lymph node metastases confirms tumour promotion in later stages and the relevance of (context-specific) TGF-B targeting [79]. A spatial shift in TGF signalling, for example from tumour to stroma with hijacked wound healing physiology [80], might be a mechanistic explanation for the reverse biological effects of TGF-B in late-stage disease.

We could not objectify the typical “geometry” of EMT, such as less nuclear roundness, in our cohort. This is partly surprising, as we had expected a more spindle-shaped morphology in more aggressive PDAC, as nuclei become more elongated in mesenchymal-type cells [81, 82]. Given the recent studies showing pronounced EMT in the TF, we would have expected a more distinct EMT phenotype [67, 83]. Nevertheless, multiple other factors, e.g. tissue pressure [84] and cellular migration [85], can shape nuclear morphology which we do not consider here, as they are not easily quantifiable. Recent evidence supports an “amoeboid” PDAC phenotype within the EMT spectrum, associated with disease progression and enriched at the invasive front, which retains conventional cellular geometry and appears TGF-β-induced [86–88]. Contrary to previous works (e.g [31, 89])., the proportion of stroma content per tissue core did not correlate with overall survival in our study. The alignment of collagen fibrils, matrix stiffness, the stromal composition [90–92] and the local immune context [93] might outweigh the mere amount of stroma in terms of negative prognosis [94, 95]. The stroma itself is dynamic [96] and susceptible to various microenvironmental cues, and dense desmoplasia alone does not impede T-cell infiltration [97]. Limitations of our study include a relatively small sample size, which is also a limitation of the TCGA validation. In addition, not all patients were represented with three tissue cores, and some cores were lost due to deep TMA sectioning. Additionally, core exclusion was necessary because of the rather low number of tumour cells per core due to infiltrative growth, low tumour cell density, and the strong desmoplastic reaction typical of PDAC. To address discrepancies in expression due to intratumoural heterogeneity, “topographic” TMAs have been proposed [98]. These capture a multitude of biological regions of interest, such as desmoplasia, inflammation and vascular invasion. The uniformly high biological aggressiveness of PDAC, characterized by vascular and perineural invasion in most tumours (e.g., only *n* = 3 non-neoadjuvant patients without perineural invasion), limited the power of group comparisons. In a few cases, the classifier showed inconsistent performance in identifying single cells and in detecting poorly formed carcinoma glands and areas of tumour budding. Although multiple markers of interest were analysed, each analysis was performed single-plex, using different sections, which affected the spatial analysis. Deeper insights into the true immune-stroma-cancer crosstalk could be achieved by more multiplexing with simultaneous visualization of RNA and proteins, e.g. by immunofluorescence on the same tissue slide. Further validation and extension of our findings would be possible through future studies using spatially resolved (multiplex) technologies at the whole tissue level.

In this work, we spatially quantified key TGF- and BMP-signalling molecules in tumour regions and the juxtatumoural stroma. We highlight the importance of stromal signalling as a prognostic factor for overall survival and corroborate ID1^high^, TGF-B2^high^ and GREM1^low^ tumours as more aggressive.

## Supplementary Information

Below is the link to the electronic supplementary material.

Supplementary Material 1. Representative examples of each marker per compartment (PDAC parenchyma and stroma). Mean stratified low and high expression; 10x. *Inset*: Region of interest.

Supplementary Material 2. pSMAD2 and TGF-B1 (neoadjuvant cases excluded). **A** Juxtatumoural stroma revealed significantly higher pSMAD2 protein expression than PDAC parenchyma. **B** In PDAC parenchyma pSMAD2 expression was significantly higher in the TC, for juxtatumoural stroma the opposite was true (TF>TC). **C** TGF-B1 mRNA transcript counts were significantly higher in PDAC parenchyma. TC: Tumour Centre; TF: Tumour Front. ****p<0.00001

Supplementary Material 3. Linear regression (neoadjuvant cases excluded). **A** Higher ID protein expression in larger tumours. **B** Higher number of positive lymph nodes (LN) in TGF-B2high PDAC (TF). **C** Regression analysis, positive LN. **D** Regression analysis, tumour size. TC: Tumour Centre, TF: Tumour Front.

Supplementary Material 4. Tumour budding. **A** Example of high tumour budding (BD3) in TGF-B1low stroma. **B** Example of low tumour budding (BD1) in TGF-B1high stroma. From left to right: H&E stain, TGF-B1 in-situ hybridisation; pan-cytokeratin (MNF116) immunohistochemistry.

Supplementary Material 5. Immune infiltrate quantification (neoadjuvant cases excluded, see also Table S5). **A** High (low) FOXP3+ immune infiltrate in TGF-B1high (TGF-B2high) PDAC. **B** CD8+ T-cell infiltrates are independent of the level of ID1 protein expression. No differences in CD163+ macrophage (C) or (D) CD68+ macrophage levels in BMP4high or -low and GREM1high or -low PDAC.

Supplementary Material 6. Other survival analysis. **A** Forest plot of a multivariate Cox model (n=88; neoadjuvant cases excluded) highlighting the prognostic relevance of high stromal TGF-A in the Tumour Front (TF). Stromal TGF-B2 (Tumour Centre, TC) is not significant. Older age, a higher number of positive lymph nodes (LN), and larger tumor size are significantly associated with higher Hazard Ratios (HR >1). **B** The relative stromal proportion per tissue core stratified by mean (TC: mean 27.7%; TF: mean 29.2%), and **C** nuclear roundness of PDAC nuclei was not significantly associated with overall survival.

Supplementary Material 7. Representative examples of Nuclear Roundness. **A** Less nuclear roundness (0.69) in the PDAC tumour centre. Desmoplasia, tumour buds and angulated glands in higher magnification (10x). **B** Rounder nuclei (0.75) in the tumour centre of another PDAC patient of the cohort (10x). The PDAC gland is more densely populated and has on average rounder nuclei. Inset: 60x.

Supplementary Material 8. Validation in TCGA mRNA data (transcripts per million). **A** Significantly higher ID1 transcript counts in PDAC. **B** Less SMAD2 transcripts in PDAC versus normal pancreas (ns). **C** Higher ID1 transcript counts in N1-stage PDAC (ns). **D** Higher TGFB2 transcript counts in N1-stage PDAC versus N0 (statistically ns, trend). **E** Binary survival analysis (high versus low transcript counts) reveals no significant differences for TGFA, -B1, and -B2. ns: non significant.

Supplementary Tables

## Data Availability

Available upon reasonable request from the corresponding author.

## Abbreviations

AI: Artificial intelligence
BMP: Bone morphogenetic protein
CAF: Cancer-associated fibroblast
DIA: Digital image analysis
EGFR: Epidermal growth factor receptor
EMT: Epithelial-mesenchymal transition
GREM1: Gremlin1
ID1: Inhibitor of differentiation protein 1
IHC: Immunohistochemistry
ISH: In-situ hybridisation
mRNA: Messenger RNA
PDAC: Pancreatic ductal adenocarcinoma
pSMAD2: PhosphoSMAD2
SMAD: Suppressor of mothers against decapentaplegic
TC: Tumour centre
TF: Tumour front
TGF: Transforming growth factor
TMA: Tissue microarray
TME: Tumor microenvironment
