# Supplementary material for: Spatially resolved analysis of TGF/BMP signalling in pancreatic ductal adenocarcinoma by digital pathology identifies patient subgroups with adverse outcome
Source: BMC Cancer. 2025 Aug 18;25:1327. doi: 10.1186/s12885-025-14751-3 (PMC12359875; doi:10.1186/s12885-025-14751-3)
Supplement: Supplementary file 1 — Supplementary Material 1. Representative examples of each marker per compartment (PDAC parenchyma and stroma). Mean stratified low and high expression; 10x. Inset: Region of interest. [file 12885_2025_14751_MOESM1_ESM.pdf]

Figure S1
PDAC parenchyma

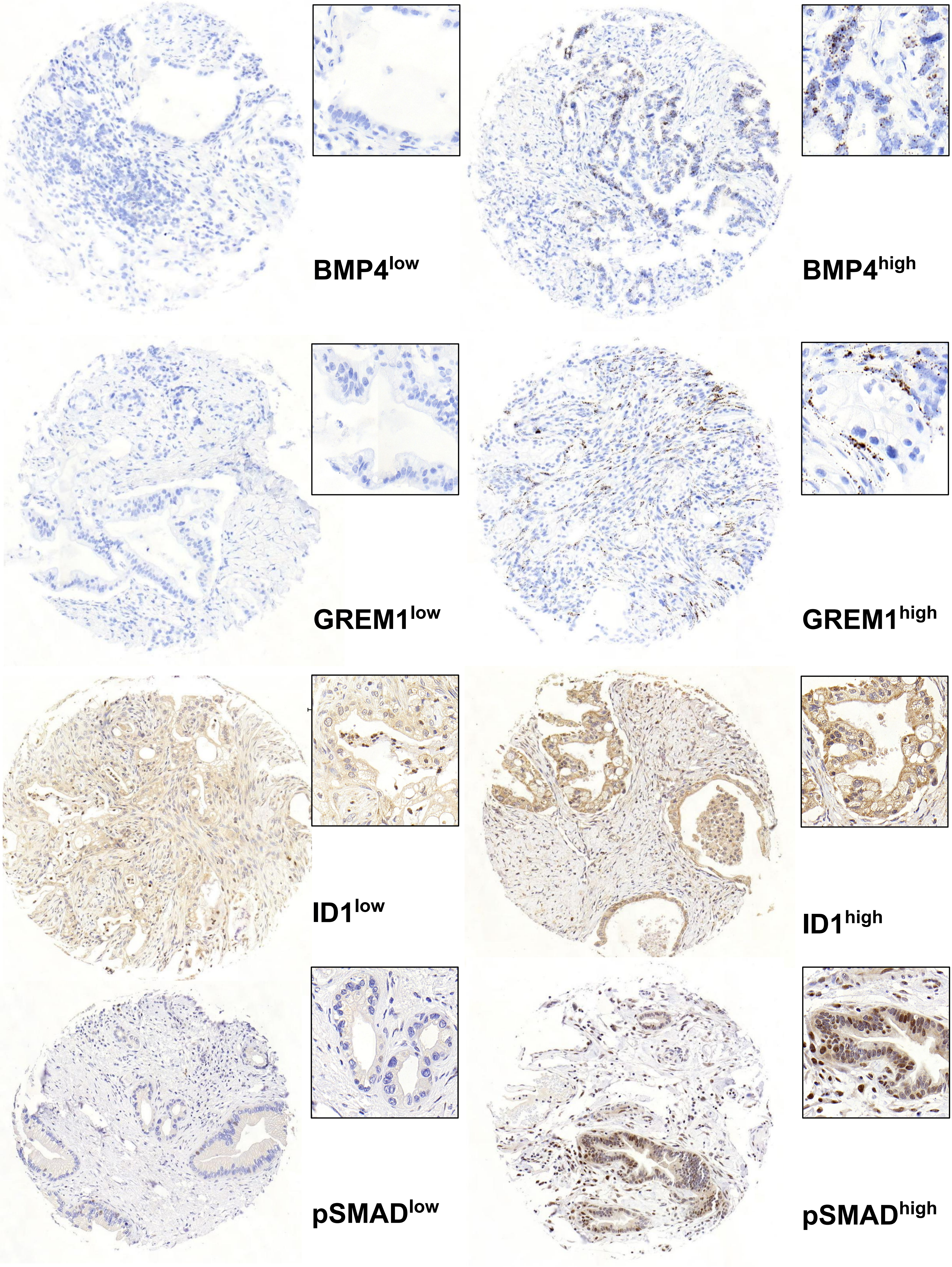

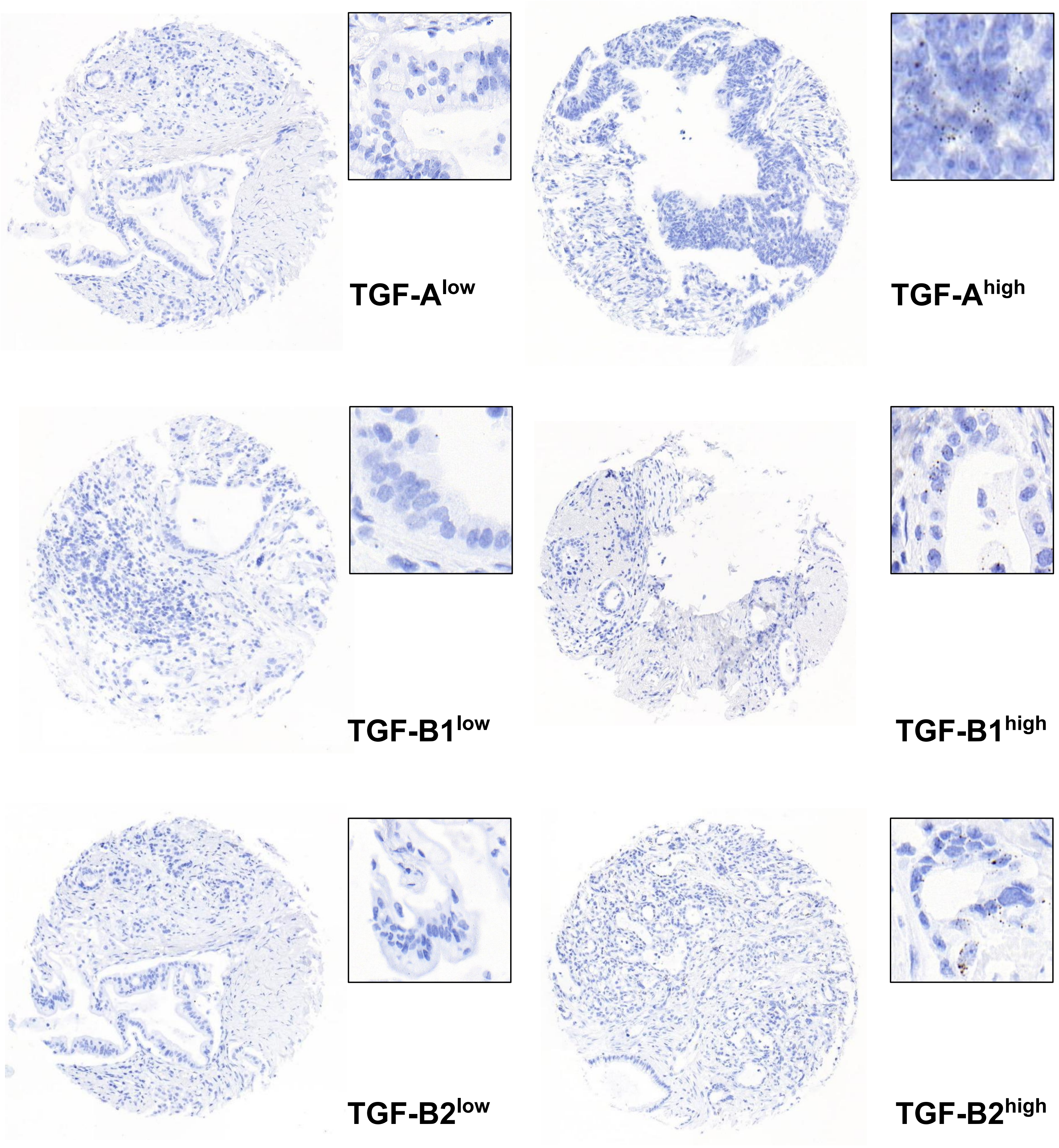

TGF-A^{low}
TGF-A^{high}
TGF-B1^{low}
TGF-B1^{high}
TGF-B2^{low}
TGF-B2^{high}

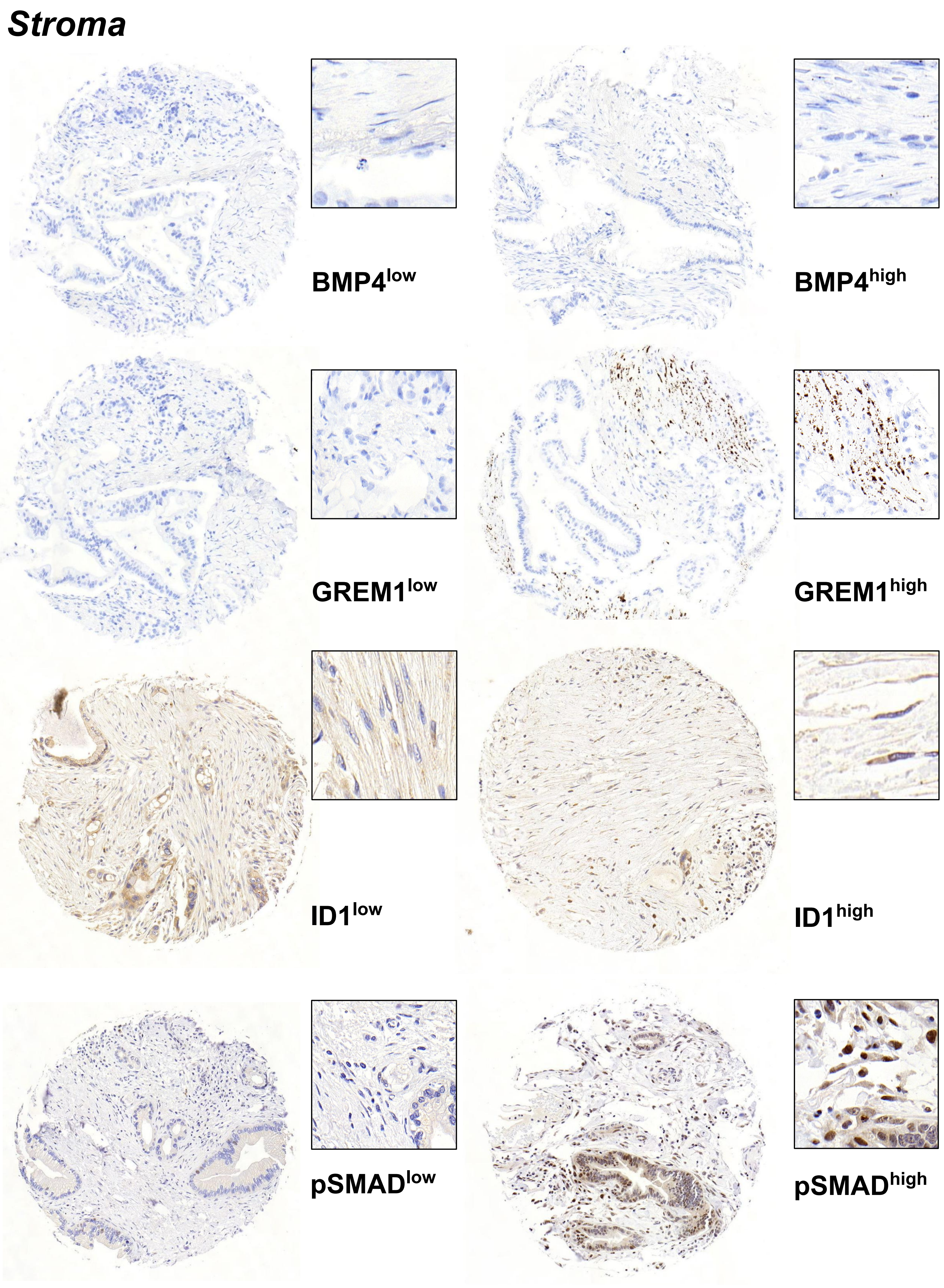
Stroma
BMP4low
BMP4high
GREM1low
GREM1high
ID1low
ID1high
pSMADlow
pSMADhigh

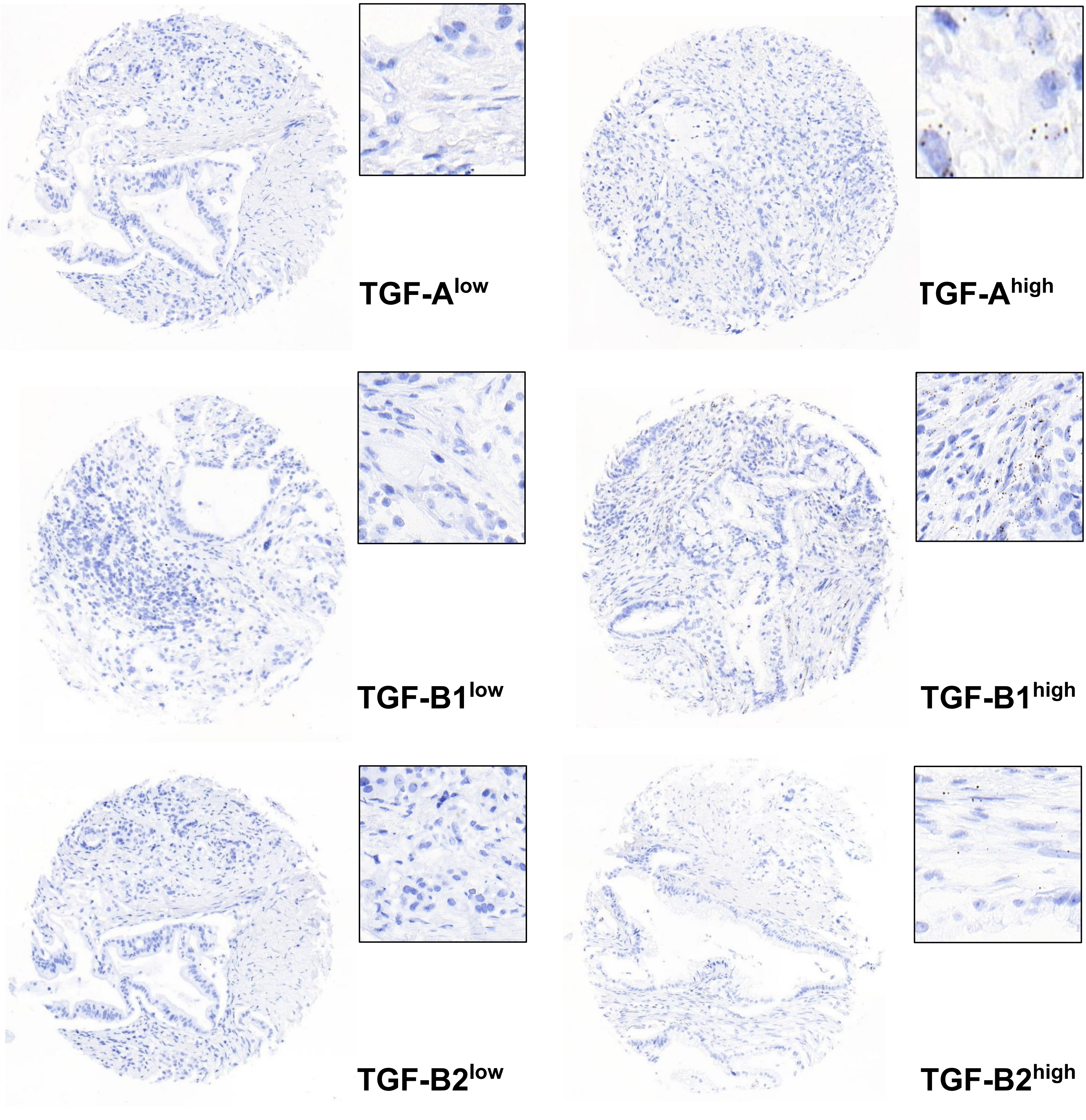
TGF-A^{low}
TGF-A^{high}
TGF-B1^{low}
TGF-B1^{high}
TGF-B2^{low}
TGF-B2^{high}
