# Supplementary figures and images for: Spatially resolved analysis of TGF/BMP signalling in pancreatic ductal adenocarcinoma by digital pathology identifies patient subgroups with adverse outcome
Source: BMC Cancer. 2025 Aug 18;25:1327. doi: 10.1186/s12885-025-14751-3 (PMC12359875; doi:10.1186/s12885-025-14751-3)

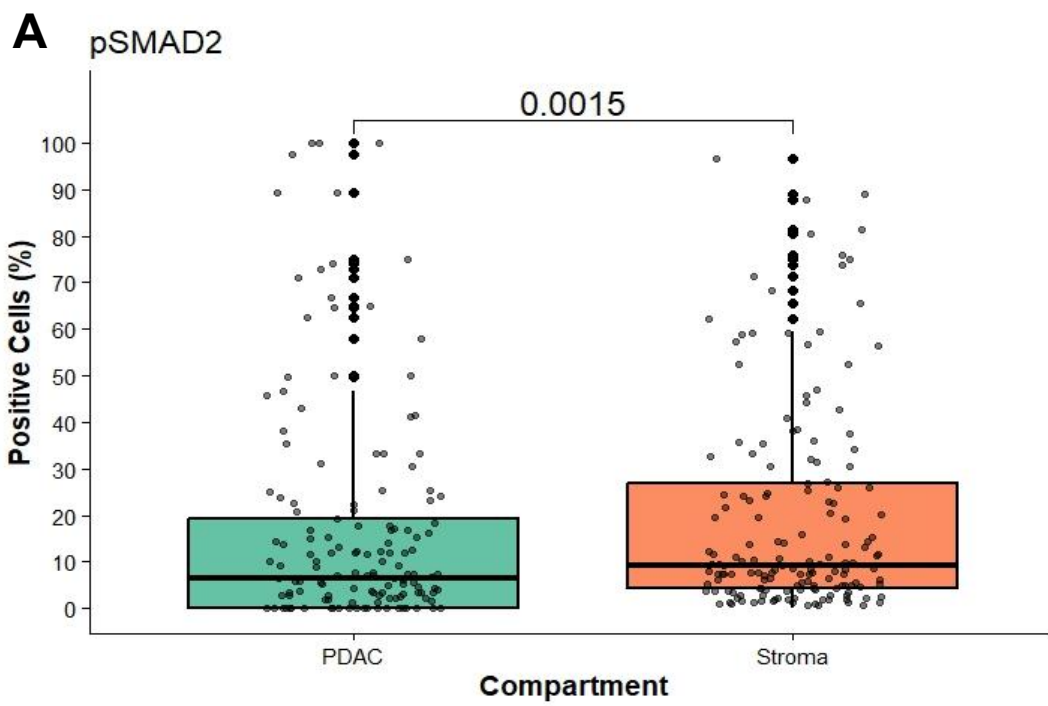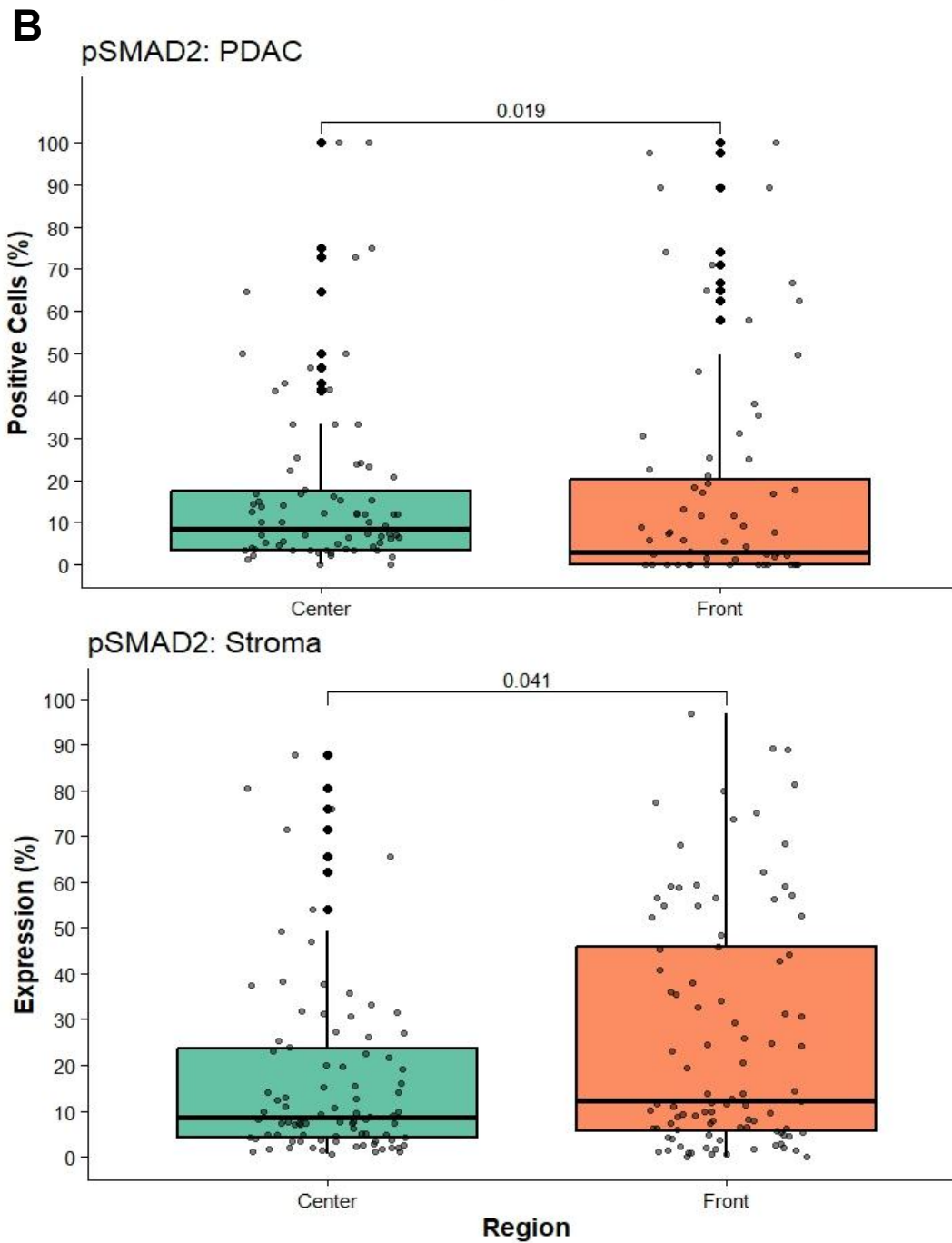

**C**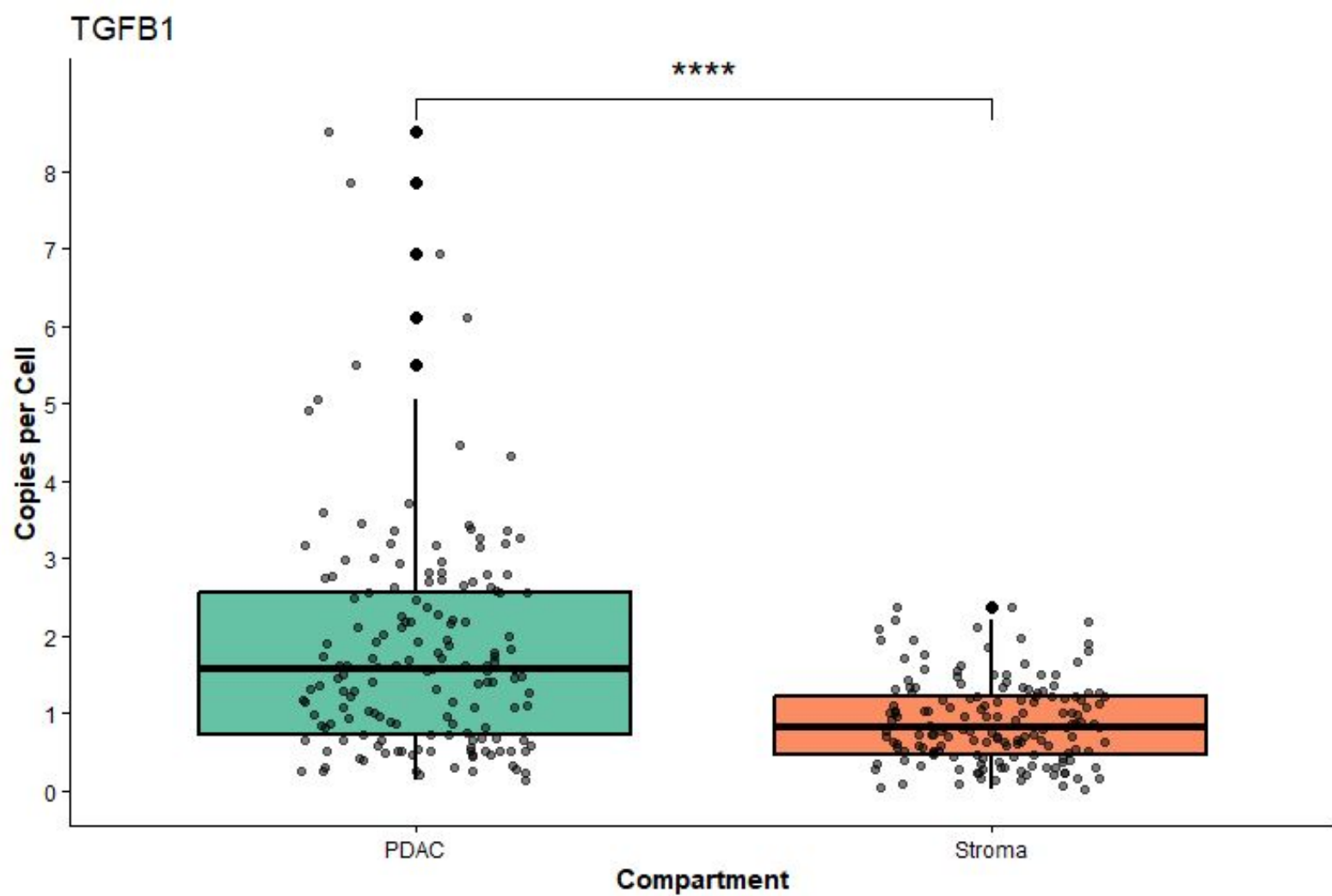

Supplement: Supplementary file 2 — Supplementary Material 2. pSMAD2 and TGF-B1 (neoadjuvant cases excluded). A Juxtatumoural stroma revealed significantly higher pSMAD2 protein expression than PDAC parenchyma. B In PDAC parenchyma pSMAD2 expression was significantly higher in the TC, for juxtatumoural stroma the opposite was true (TF>TC). C TGF-B1 mRNA transcript counts were significantly higher in PDAC parenchyma. TC: Tumour Centre; TF: Tumour Front. ****p<0.00001 [file 12885_2025_14751_MOESM2_ESM.pdf]

**A**

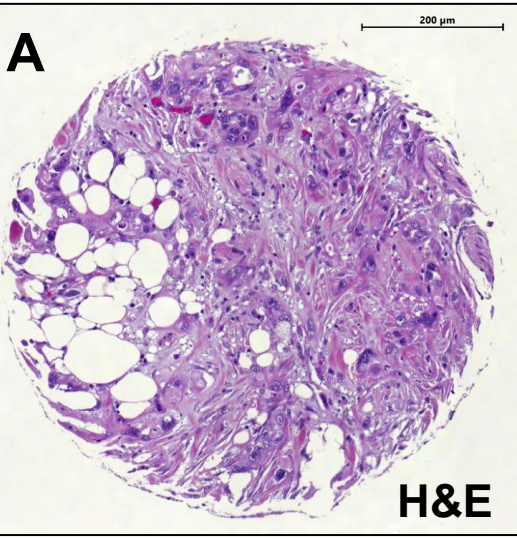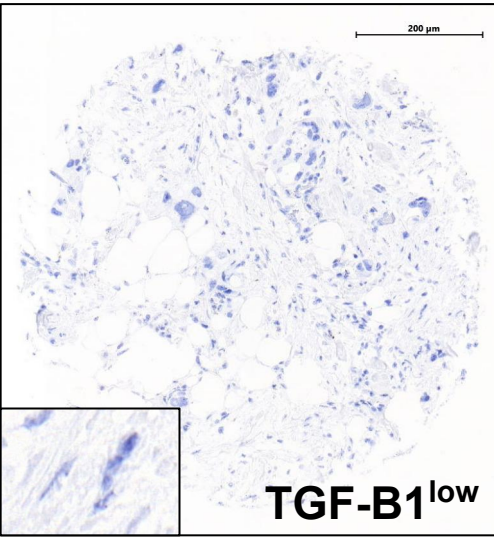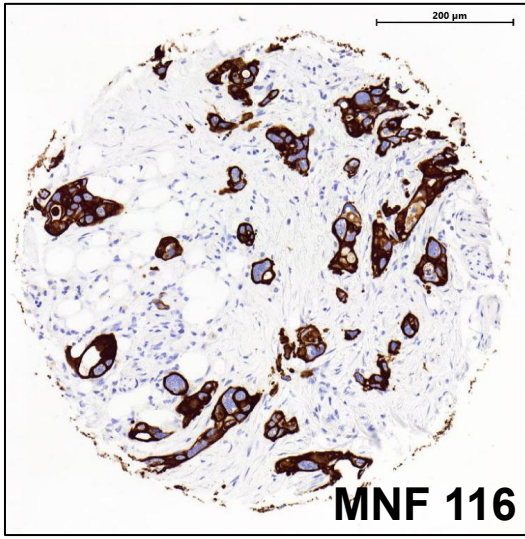

**B**

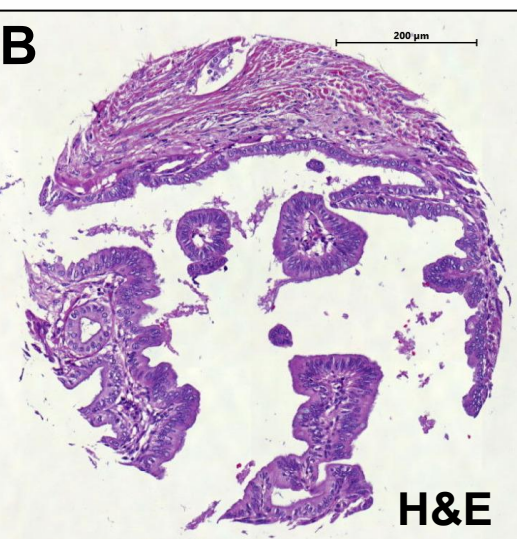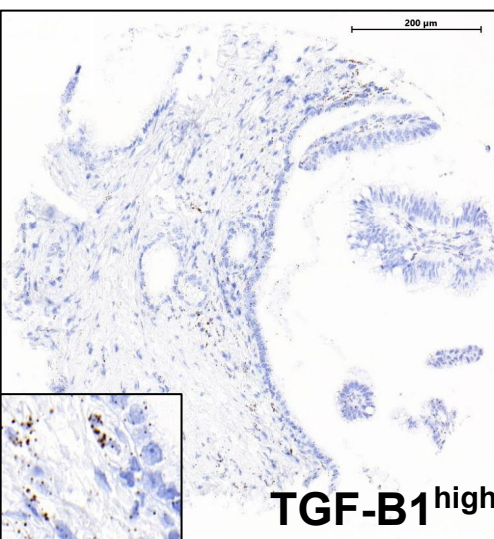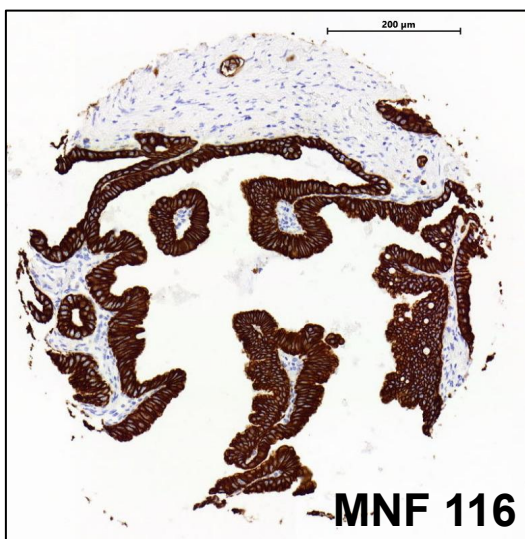

Supplement: Supplementary file 4 — Supplementary Material 4. Tumour budding. A Example of high tumour budding (BD3) in TGF-B1low stroma. B Example of low tumour budding (BD1) in TGF-B1high stroma. From left to right: H&E stain, TGF-B1 in-situ hybridisation; pan-cytokeratin (MNF116) immunohistochemistry. [file 12885_2025_14751_MOESM4_ESM.pdf]

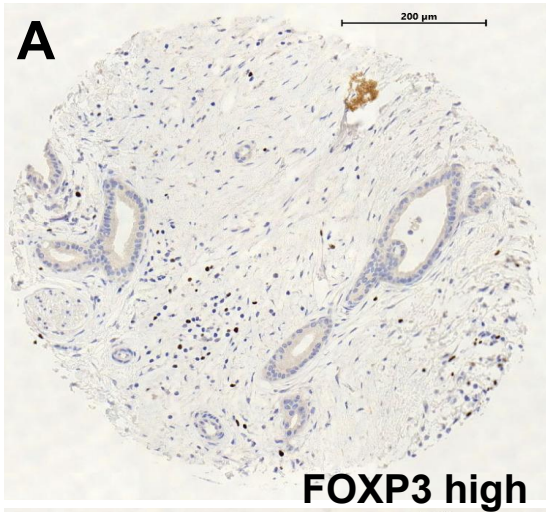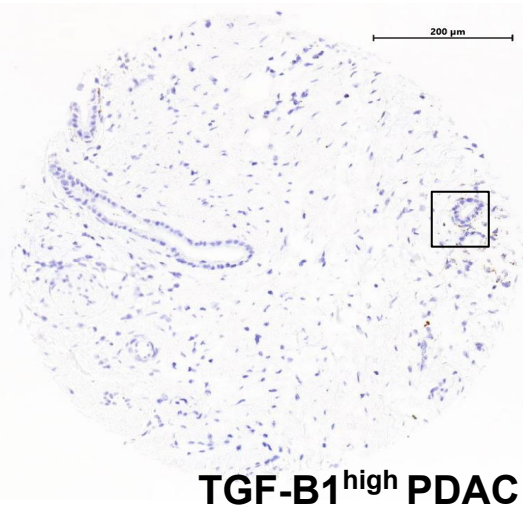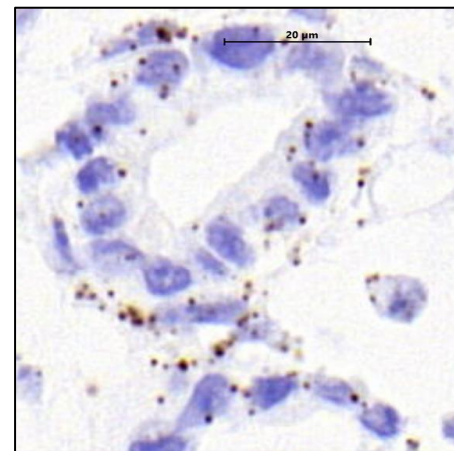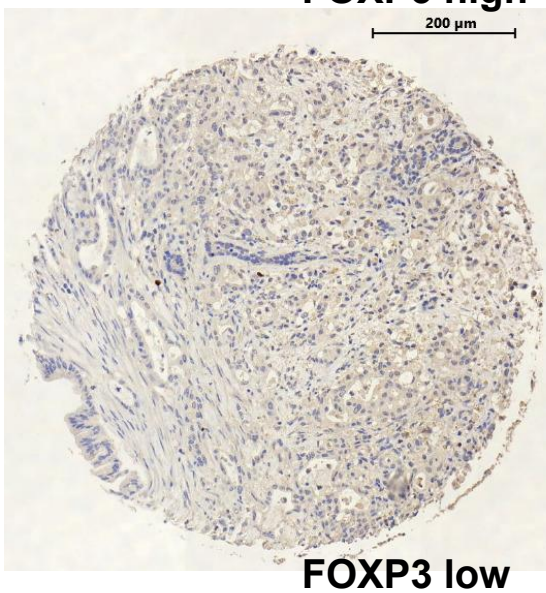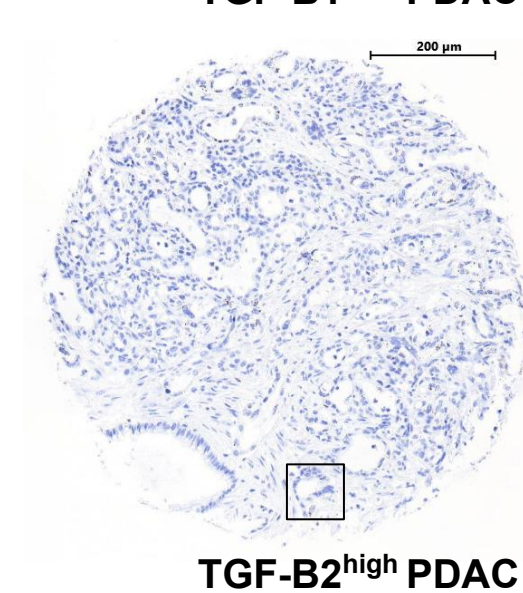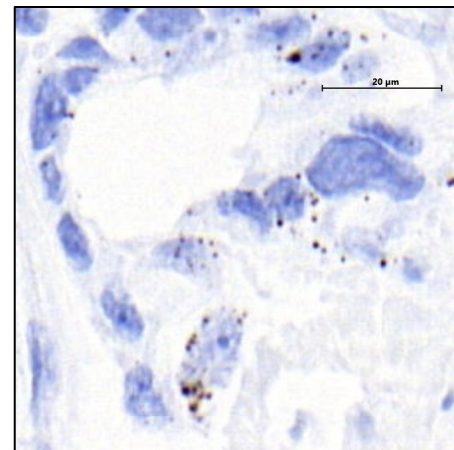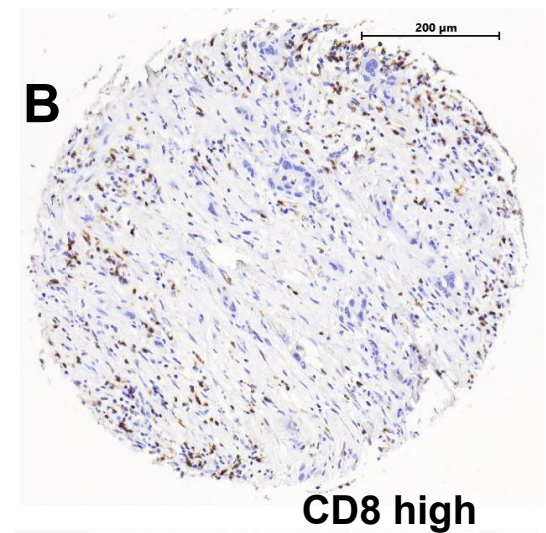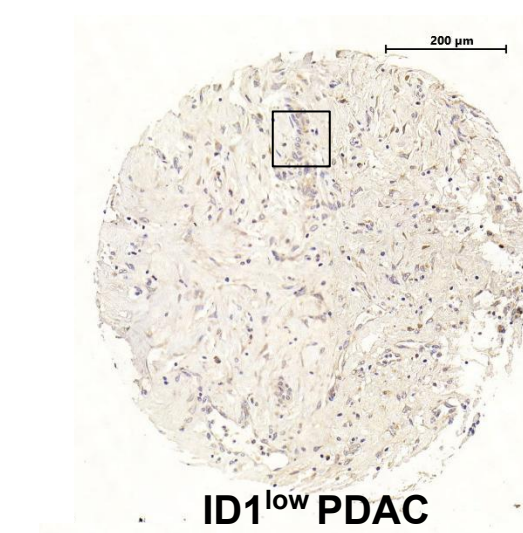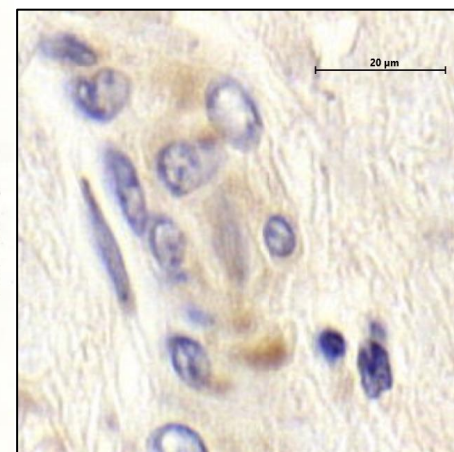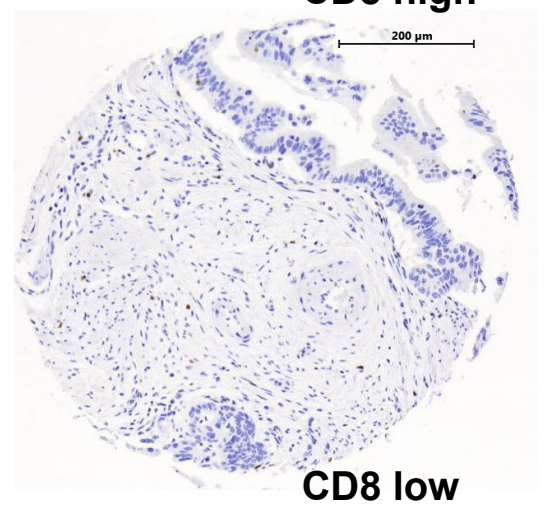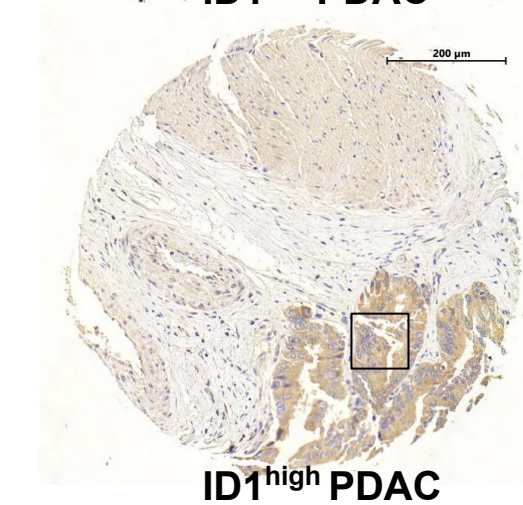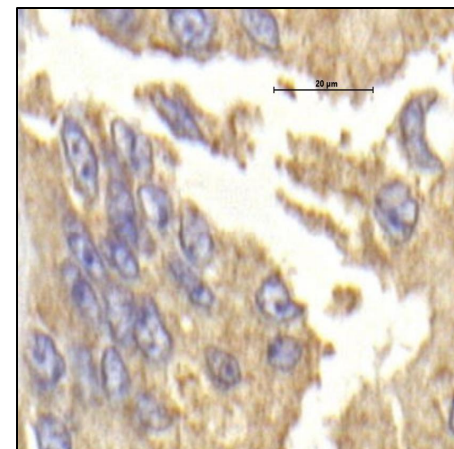

**C**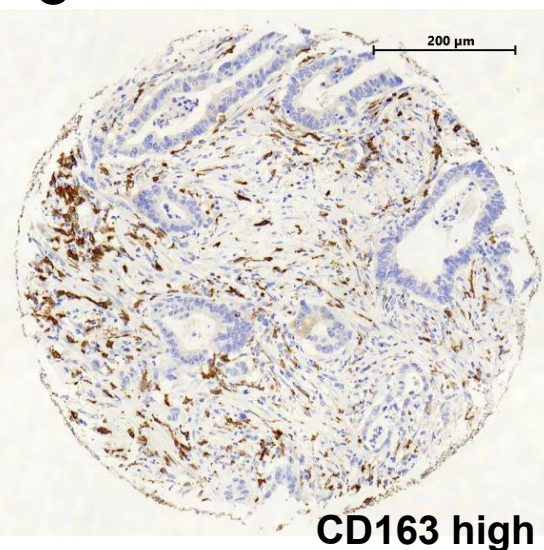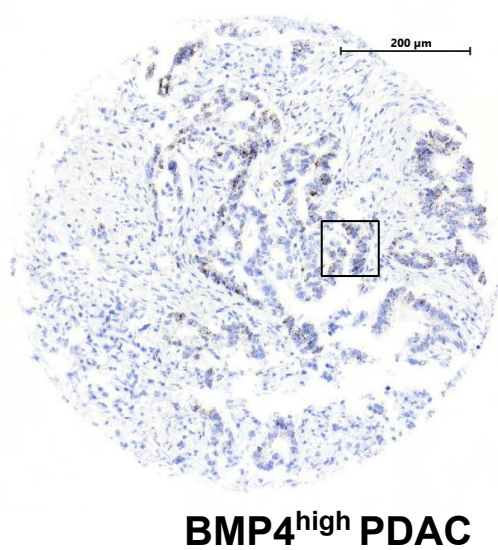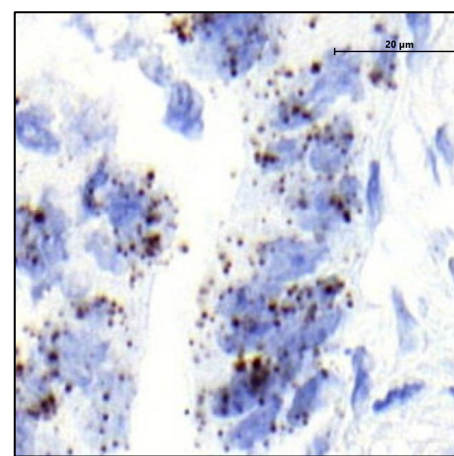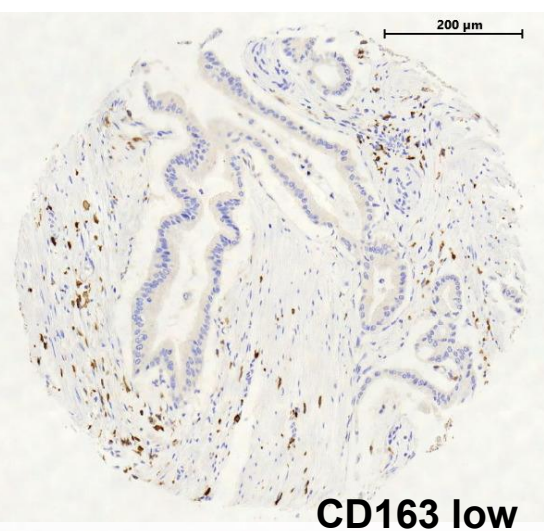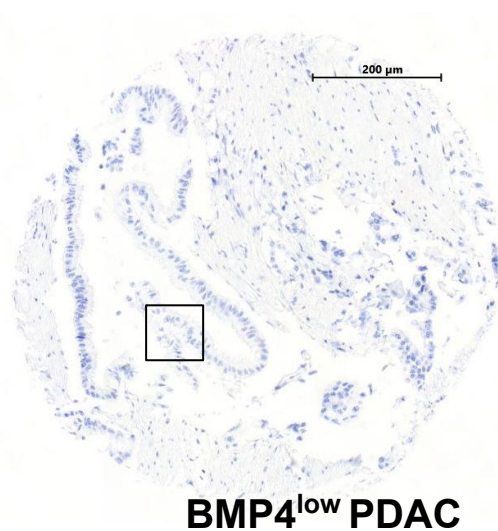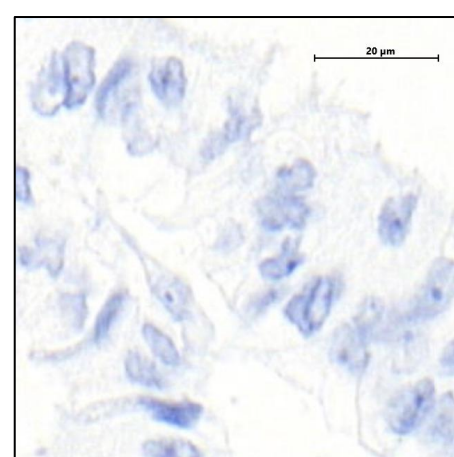**D**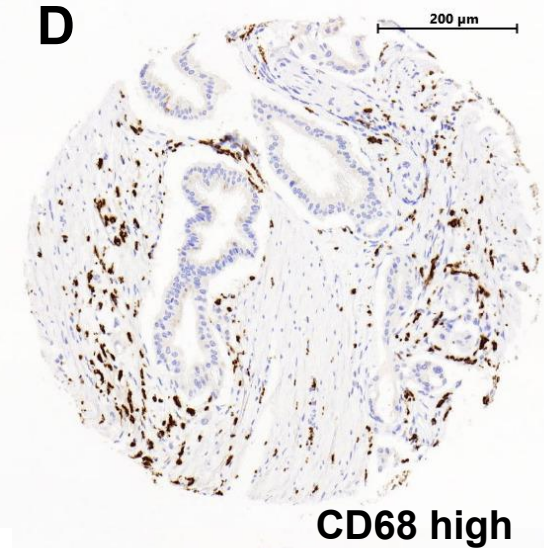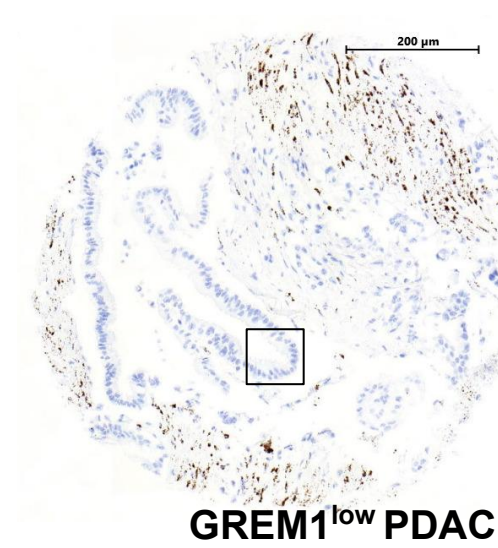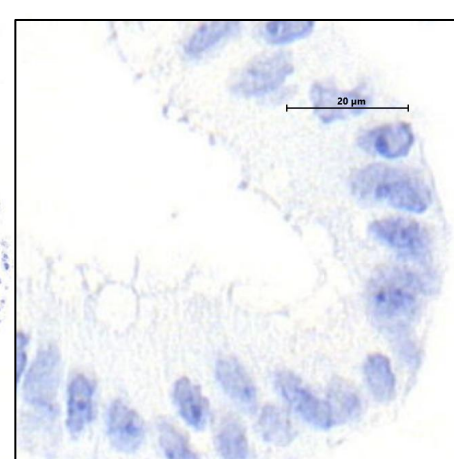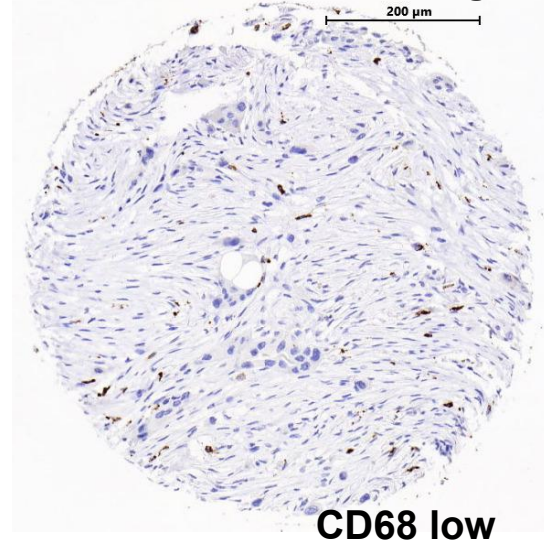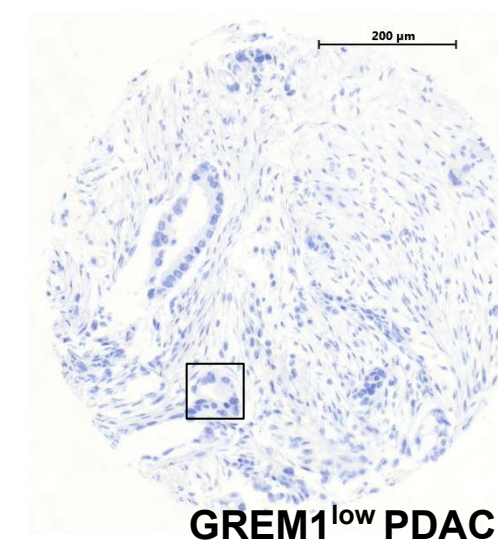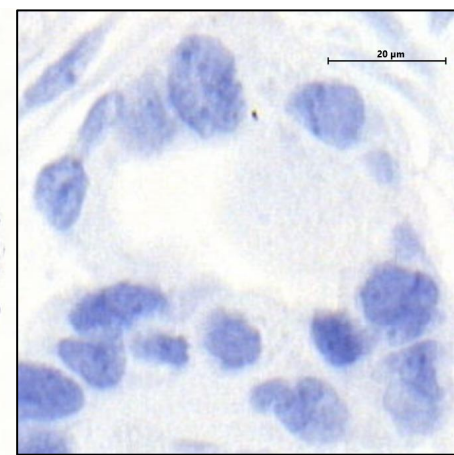

Supplement: Supplementary file 5 — Supplementary Material 5. Immune infiltrate quantification (neoadjuvant cases excluded, see also Table S5). A High (low) FOXP3+ immune infiltrate in TGF-B1high (TGF-B2high) PDAC. B CD8+ T-cell infiltrates are independent of the level of ID1 protein expression. No differences in CD163+ macrophage (C) or (D) CD68+ macrophage levels in BMP4high or -low and GREM1high or -low PDAC. [file 12885_2025_14751_MOESM5_ESM.pdf]

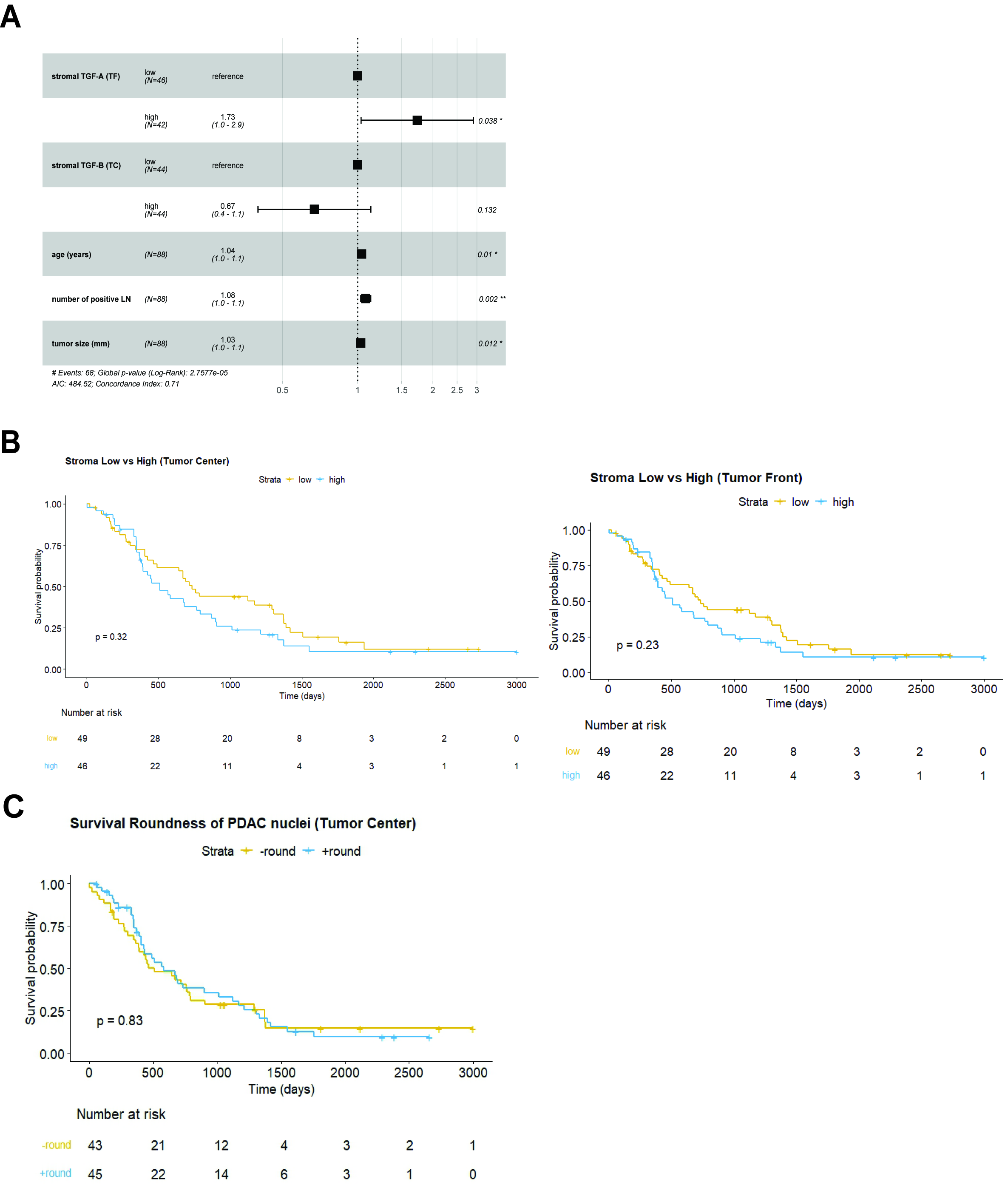

Supplement: Supplementary file 6 — Supplementary Material 6. Other survival analysis. A Forest plot of a multivariate Cox model (n=88; neoadjuvant cases excluded) highlighting the prognostic relevance of high stromal TGF-A in the Tumour Front (TF). Stromal TGF-B2 (Tumour Centre, TC) is not significant. Older age, a higher number of positive lymph nodes (LN), and larger tumor size are significantly associated with higher Hazard Ratios (HR >1). B The relative stromal proportion per tissue core stratified by mean (TC: mean 27.7%; TF: mean 29.2%), and C nuclear roundness of PDAC nuclei was not significantly associated with overall survival. [file 12885_2025_14751_MOESM6_ESM.tif]

**A**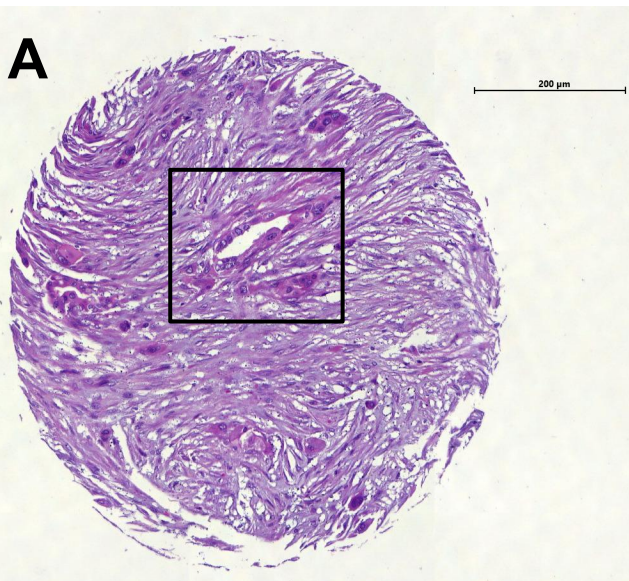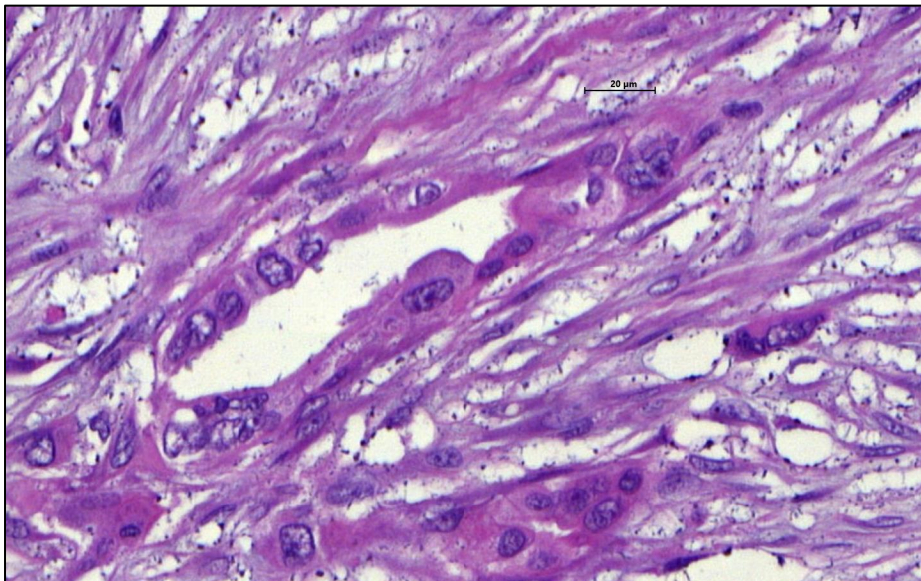**B**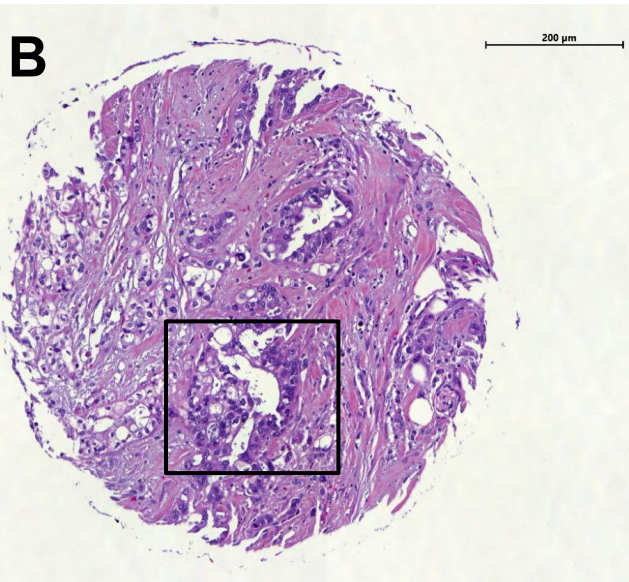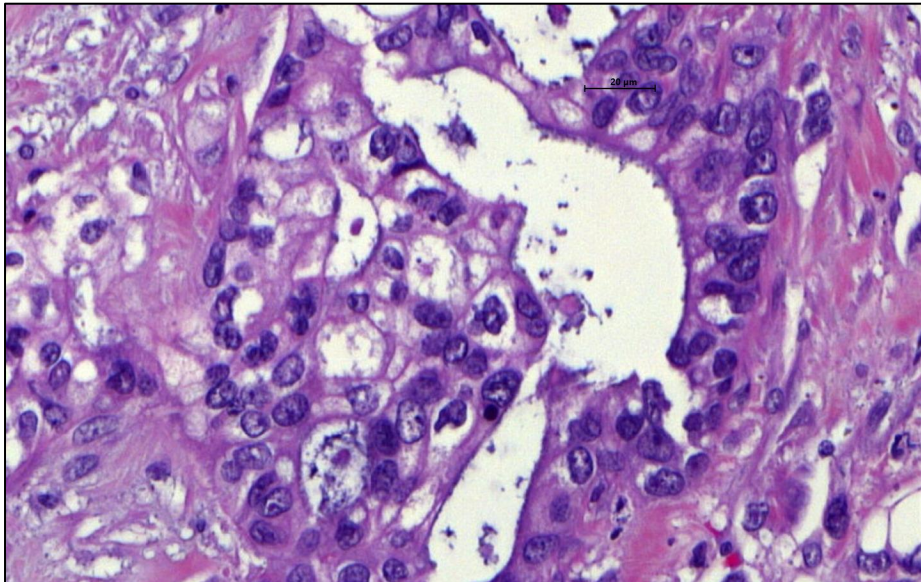

Supplement: Supplementary file 7 — Supplementary Material 7. Representative examples of Nuclear Roundness. A Less nuclear roundness (0.69) in the PDAC tumour centre. Desmoplasia, tumour buds and angulated glands in higher magnification (10x). B Rounder nuclei (0.75) in the tumour centre of another PDAC patient of the cohort (10x). The PDAC gland is more densely populated and has on average rounder nuclei. Inset: 60x. [file 12885_2025_14751_MOESM7_ESM.pdf]

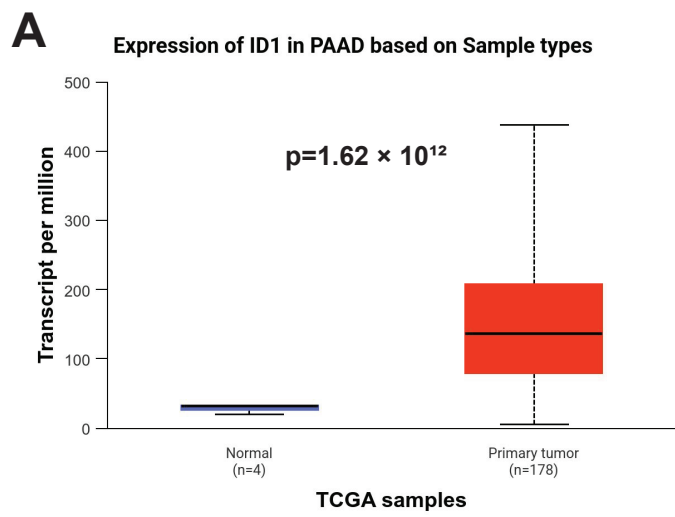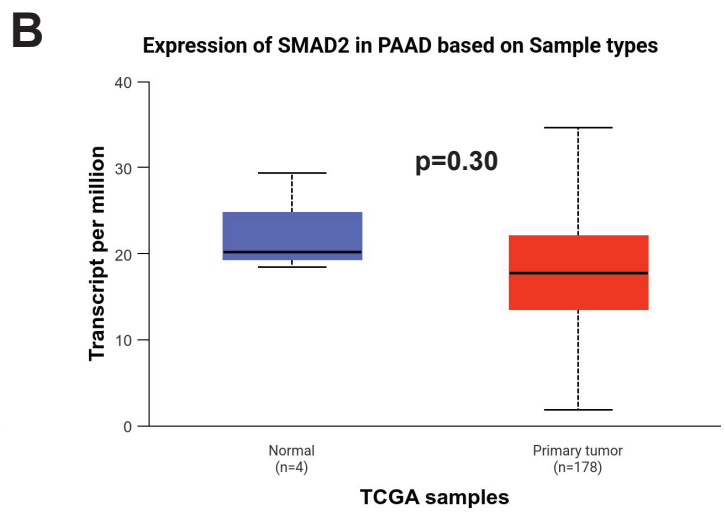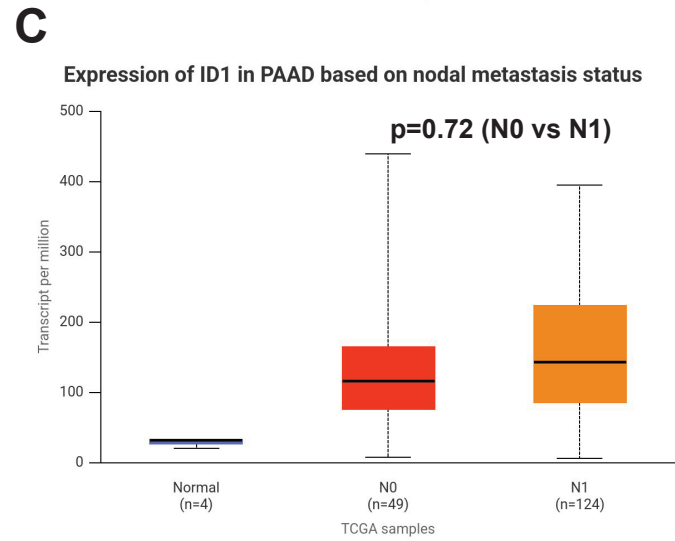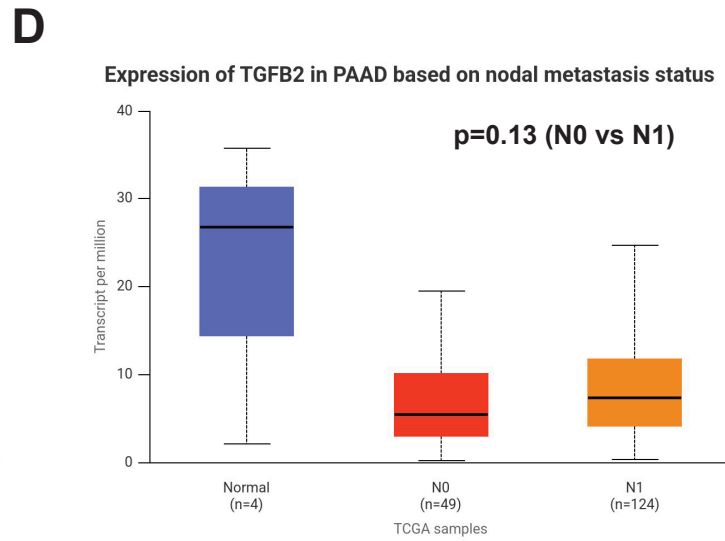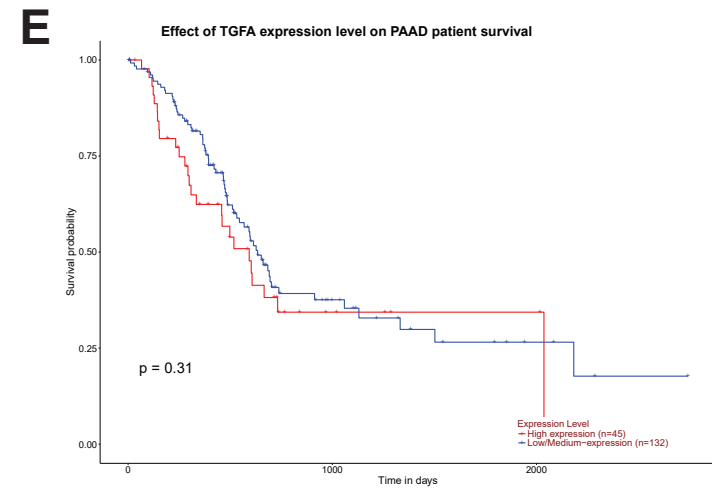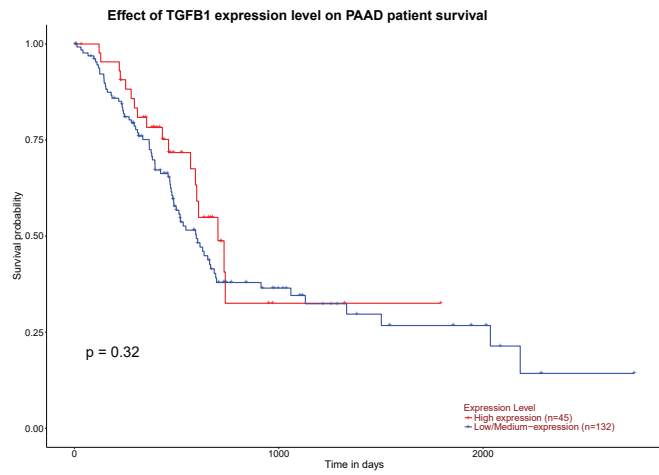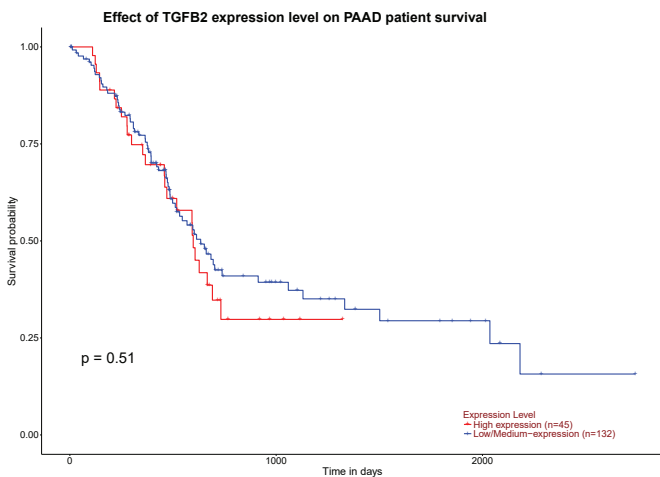

Supplement: Supplementary file 8 — Supplementary Material 8. Validation in TCGA mRNA data (transcripts per million). A Significantly higher ID1 transcript counts in PDAC. B Less SMAD2 transcripts in PDAC versus normal pancreas (ns). C Higher ID1 transcript counts in N1-stage PDAC (ns). D Higher TGFB2 transcript counts in N1-stage PDAC versus N0 (statistically ns, trend). E Binary survival analysis (high versus low transcript counts) reveals no significant differences for TGFA, -B1, and -B2. ns: non significant. [file 12885_2025_14751_MOESM8_ESM.pdf]
