# Supplementary material for: Spatially resolved analysis of TGF/BMP signalling in pancreatic ductal adenocarcinoma by digital pathology identifies patient subgroups with adverse outcome
Source: BMC Cancer. 2025 Aug 18;25:1327. doi: 10.1186/s12885-025-14751-3 (PMC12359875; doi:10.1186/s12885-025-14751-3)
Supplement: Supplementary file 3 — Supplementary Material 3. Linear regression (neoadjuvant cases excluded). A Higher ID protein expression in larger tumours. B Higher number of positive lymph nodes (LN) in TGF-B2high PDAC (TF). C Regression analysis, positive LN. D Regression analysis, tumour size. TC: Tumour Centre, TF: Tumour Front. [file 12885_2025_14751_MOESM3_ESM.pdf]

A

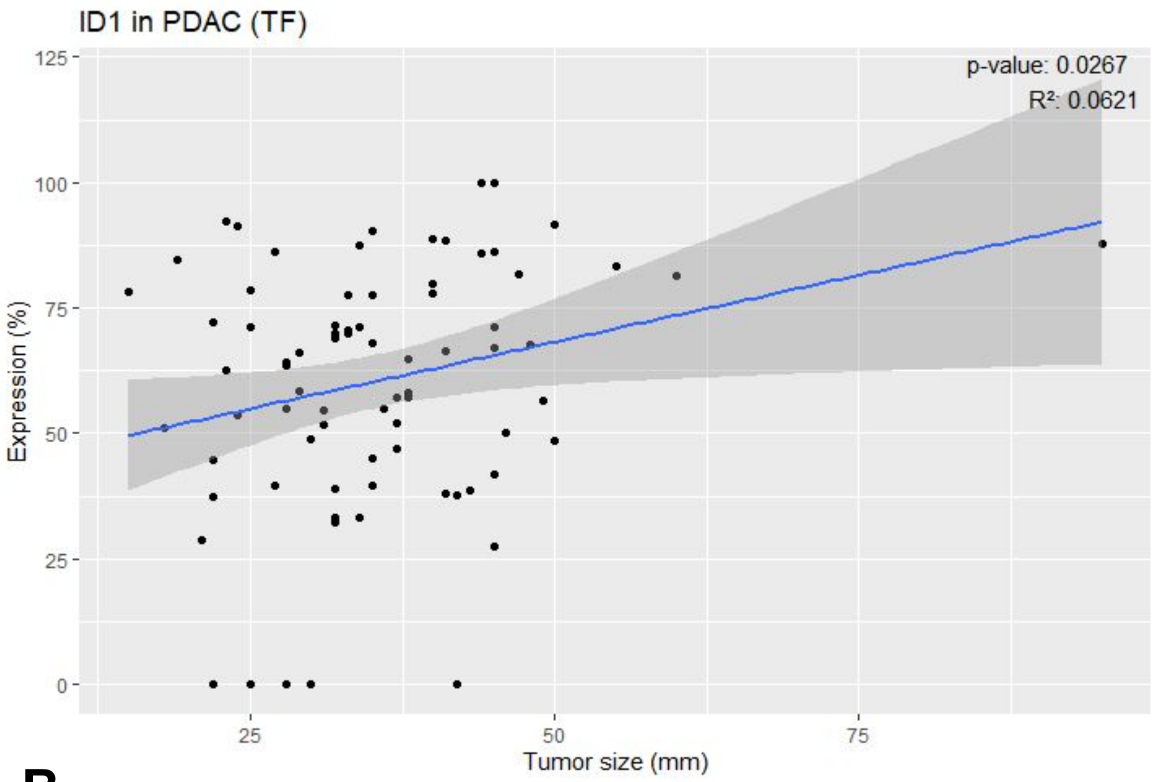

ID1 in PDAC (TF)
p-value: 0.0267
R²: 0.0621
Expression (%)
125
100
75
50
25
0
25
50
75
Tumor size (mm)

B

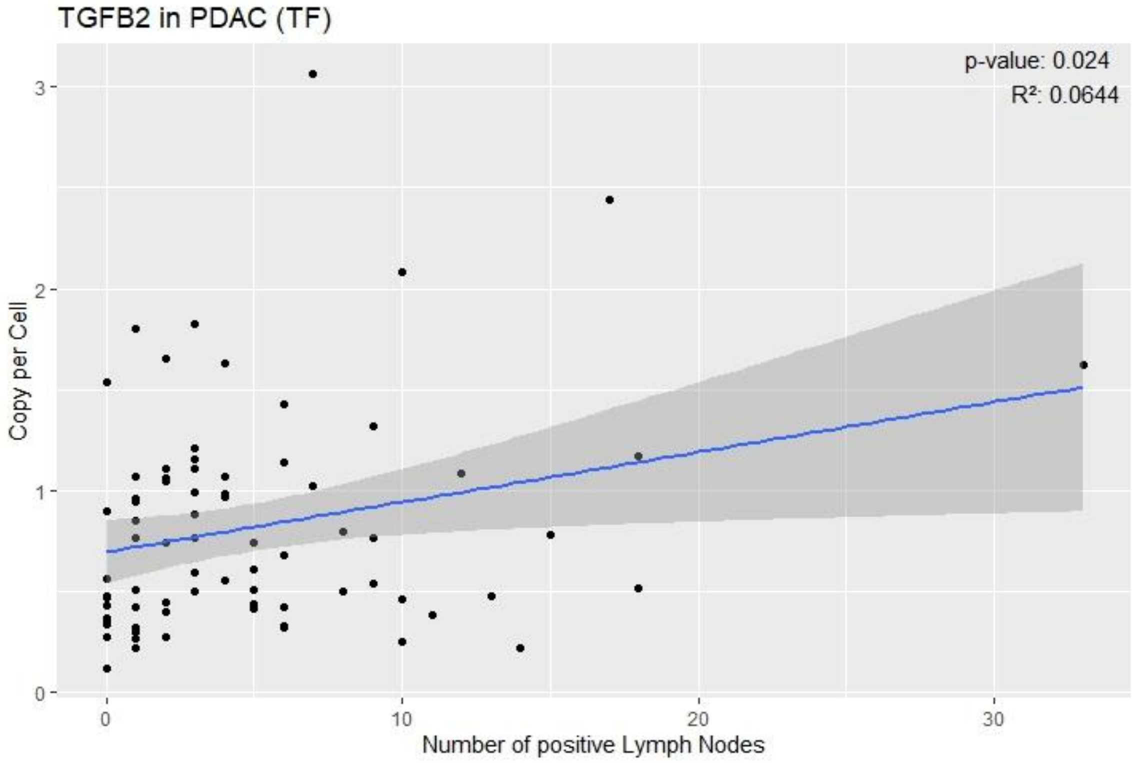

TGFB2 in PDAC (TF)
p-value: 0.024
R²: 0.0644
Copy per Cell
3
2
1
0
0
10
20
30
Number of positive Lymph Nodes

C

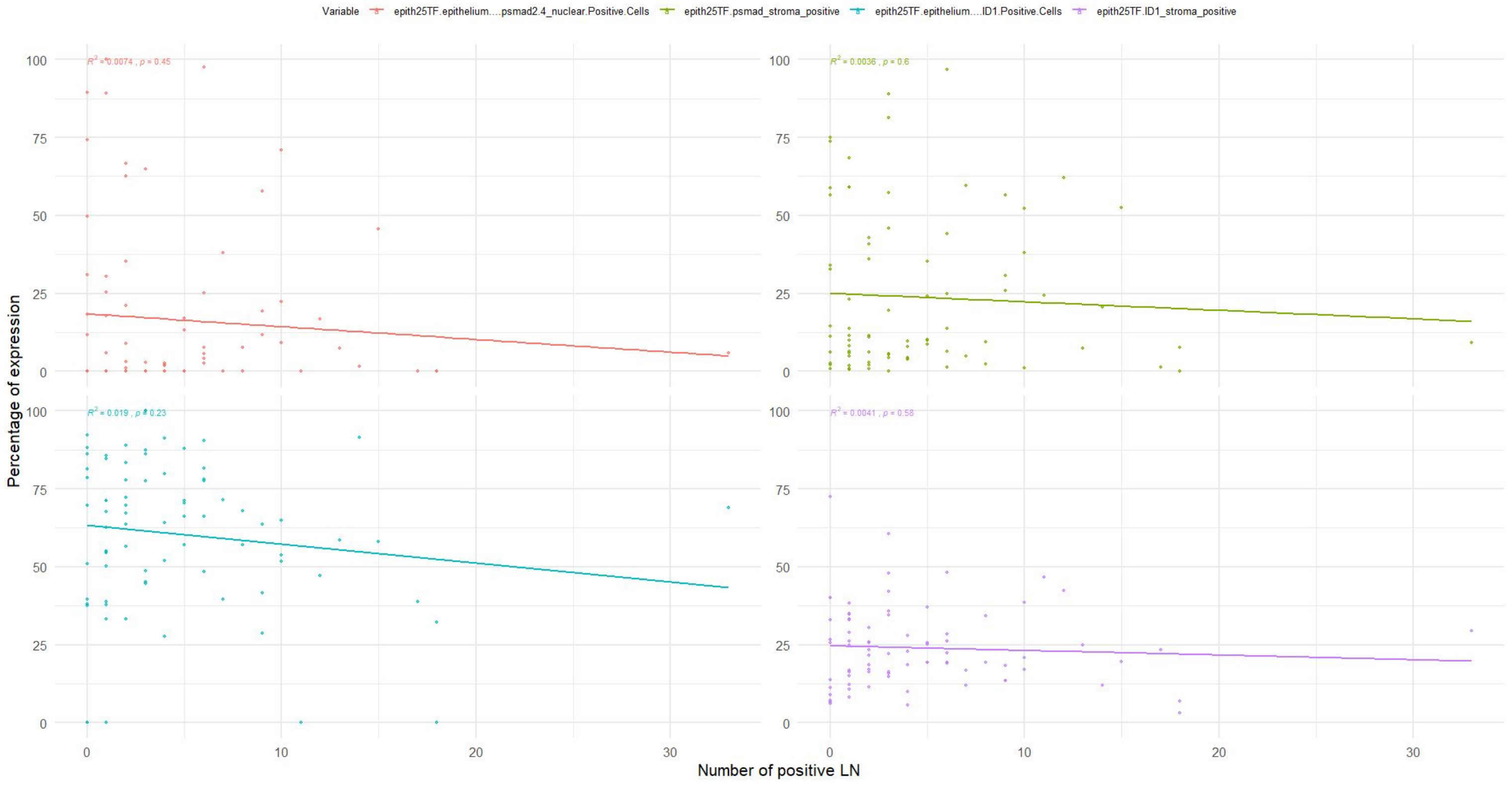
Variable
epith25TF.epithelium....psmad2.4_nuclear.Positive.Cells
epith25TF.psmad_stroma_positive
epith25TF.epithelium....ID1.Positive.Cells
epith25TF.ID1_stroma_positive
R^2 = 0.0074 , p = 0.45
R^2 = 0.0036 , p = 0.6
R^2 = 0.019 , p = 0.23
R^2 = 0.0041 , p = 0.58
Percentage of expression
Number of positive LN

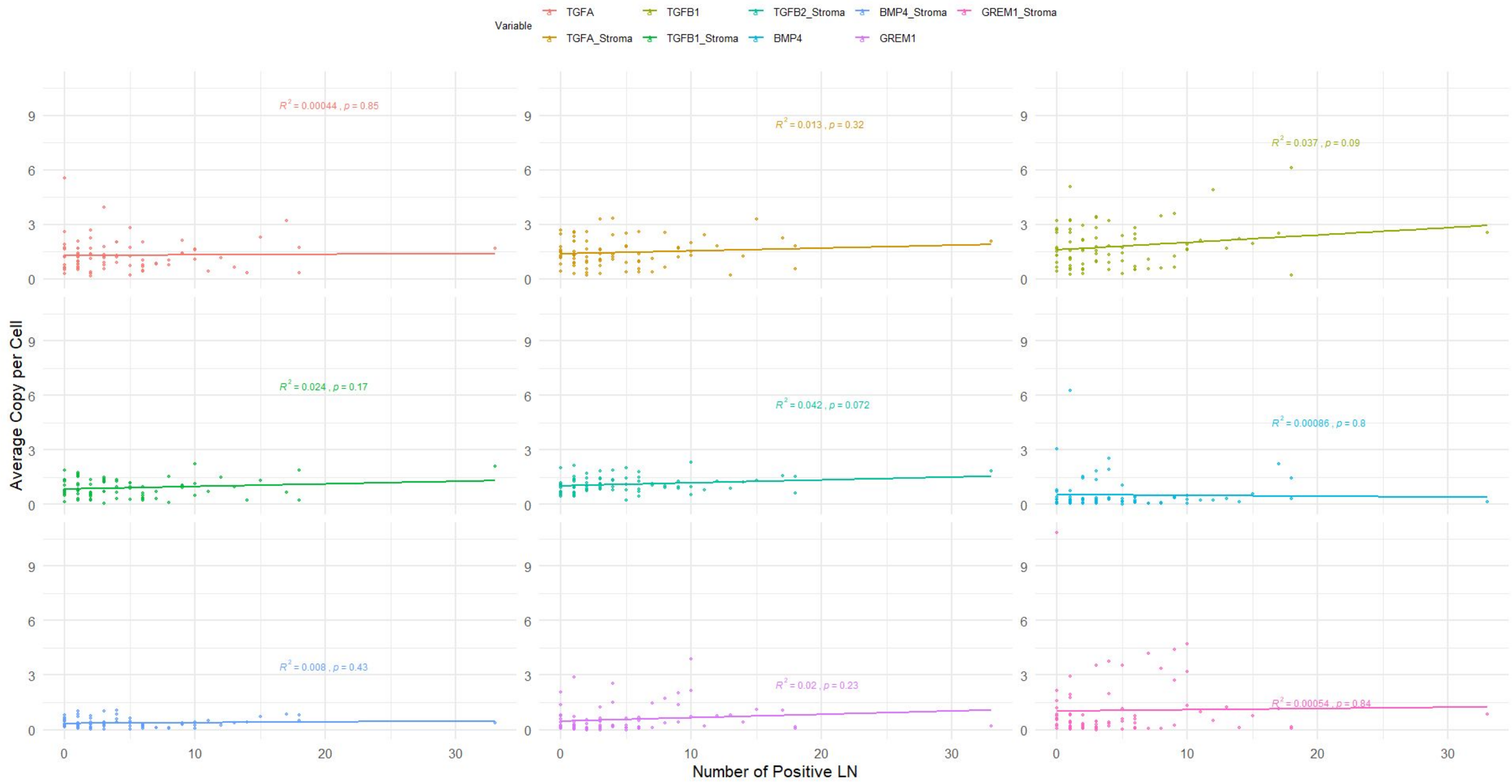
Variable
TGFA
TGFA_Stroma
TGFB1
TGFB1_Stroma
TGFB2_Stroma
BMP4
BMP4_Stroma
GREM1
GREM1_Stroma
R^2 = 0.00044 , p = 0.85
R^2 = 0.013 , p = 0.32
R^2 = 0.037 , p = 0.09
R^2 = 0.024 , p = 0.17
R^2 = 0.042 , p = 0.072
R^2 = 0.00086 , p = 0.8
R^2 = 0.008 , p = 0.43
R^2 = 0.02 , p = 0.23
R^2 = 0.00054 , p = 0.84
Average Copy per Cell
Number of Positive LN

D

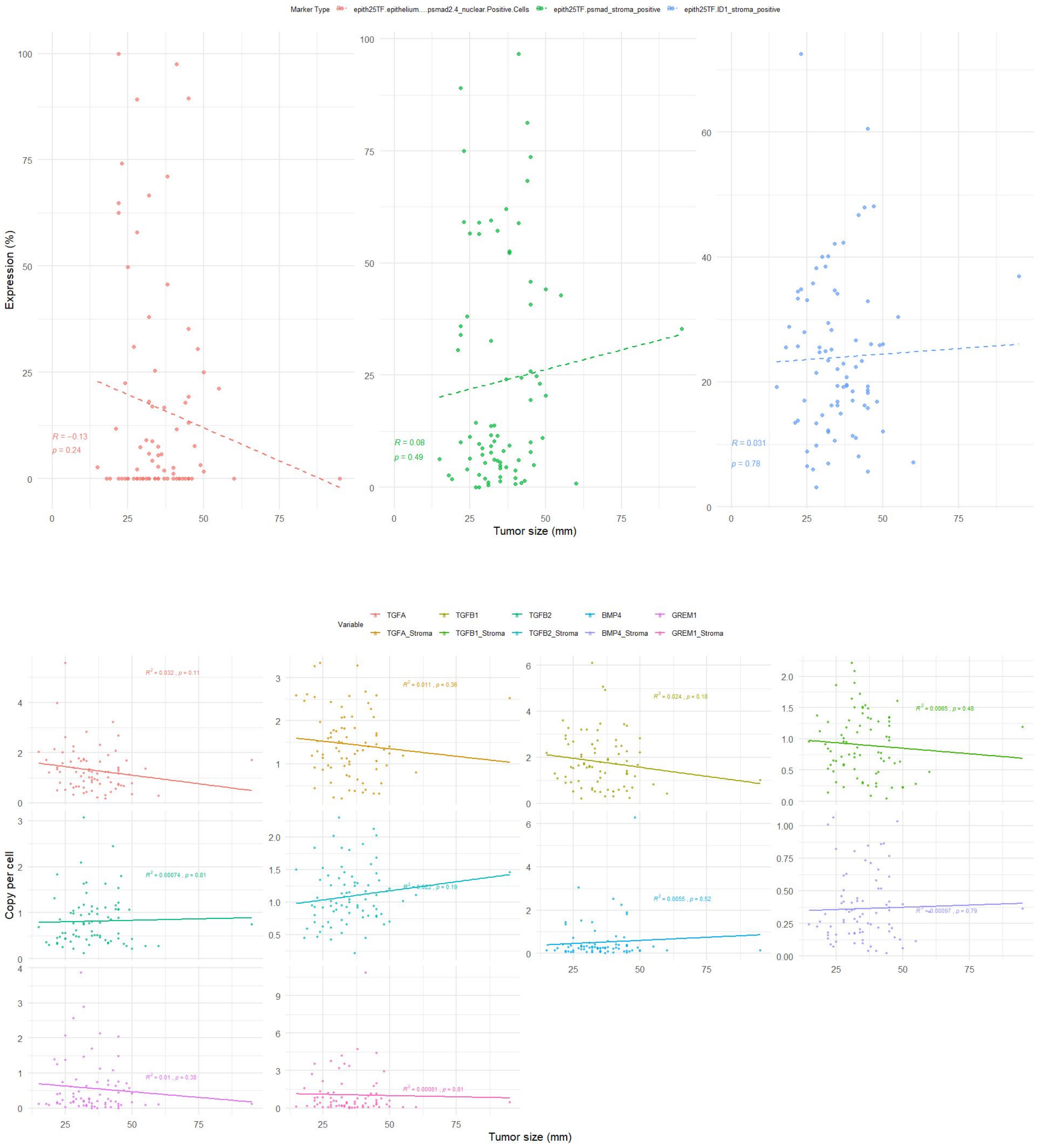

Marker Type
epith25TF.epithelium....psmad2.4_nuclear.Positive.Cells
epith25TF.psmad_stroma_positive
epith25TF.ID1_stroma_positive
Expression (%)
R = −0.13
p = 0.24
R = 0.08
p = 0.49
R = 0.031
p = 0.78
Tumor size (mm)
Variable
TGFA
TGFB1
TGFB2
BMP4
GREM1
TGFA_Stroma
TGFB1_Stroma
TGFB2_Stroma
BMP4_Stroma
GREM1_Stroma
R^2 = 0.032 , p = 0.11
R^2 = 0.011 , p = 0.36
R^2 = 0.024 , p = 0.18
R^2 = 0.0065 , p = 0.48
R^2 = 0.00074 , p = 0.81
R^2 = 0.022 , p = 0.19
R^2 = 0.0055 , p = 0.52
R^2 = 0.00097 , p = 0.79
R^2 = 0.01 , p = 0.38
R^2 = 0.00081 , p = 0.81
Copy per cell
Tumor size (mm)
