# Supplementary material for: Spatially resolved analysis of TGF/BMP signalling in pancreatic ductal adenocarcinoma by digital pathology identifies patient subgroups with adverse outcome
Source: BMC Cancer. 2025 Aug 18;25:1327. doi: 10.1186/s12885-025-14751-3 (PMC12359875; doi:10.1186/s12885-025-14751-3)
Supplement: Supplementary file 9 — Supplementary Tables [file 12885_2025_14751_MOESM9_ESM.docx]

**Supplementary Tables**

**Table S1.** Cohort details of PDAC patients who received neoadjuvant treatment (n=13). *BD*: Tumour Budding; *CAP*: College of American Pathologists; *FOLFIRINOX*: fluorouracil, irinotecan and oxaliplatin; *ITBCC*: International Tumour Budding Consensus Conference; *IPMN*: Intraductal Papillary Mucinous Neoplasm; *NA*: not available; *PanIN*: Pancreatic Intraepithelial Neoplasia; *SD*: Standard Deviation; *UICC*: Union Internationale Contre le Cancer

| **Characteristic** | **N = 13**^1^ |
| --- | --- |
| Age (years) | 67.1 (SD 7.3) |
| Gender |  |
| Female | 4 / 13 (30.8%) |
| Male | 9 / 13 (69.2%) |
| Deceased | 12 / 13 (92.3%) |
| Overall Survival (days) | 742.2 (SD 450.0) |
| Type of Resection |  |
| Left resection | 2 / 13 (15.4%) |
| Total pancreatectomy | 5 / 13 (38.5%) |
| Whipple procedure | 6 / 13 (46.2%) |
| Size invasive tumour (mm) | 35.6 (SD 27.5; range 3 to 95) |
| Intrapancreatic location invasive tumour |  |
| Head | 9 / 13 (69.2%) |
| Head, body and tail | 1 / 13 (7.7%) |
| Tail | 2 / 13 (15.4%) |
| Tail and body | 1 / 13 (7.7%) |
| T-Stage (UICC 8th) |  |
| y1a | 1 / 13 (7.7%) |
| y1c | 2 / 13 (15.4%) |
| y2 | 8 / 13 (61.5%) |
| y3 | 2 / 13 (15.4%) |
| N-Stage (UICC 8th) |  |
| y0 | 6 / 13 (46.2%) |
| y1 | 4 / 13 (30.8%) |
| y2 | 3 / 13 (23.1%) |
| M-Stage (UICC 8th) |  |
| 0 | 13 / 13 (100.0%) |
| Lymphatic vessel infiltration (L1) | 9 / 13 (69.2%) |
| Blood vessel infiltration (V1) | 7 / 13 (53.8%) |
| Perineural infiltration (Pn1) | 10 / 13 (76.9%) |
| Resection status (R) |  |
| 0 | 8 / 13 (61.5%) |
| 1 | 2 / 13 (15.4%) |
| x | 3 / 13 (23.1%) |
| Surgical resectability |  |
| Locally advanced | 11 / 13 (84.6%) |
| Borderline resectable | 2 / 13 (15.4%) |
| Tumour Regression Grade (Le Scodan) |  |
| 1 | 7 / 13 (53.8%) |
| 2 | 3 / 13 (23.1%) |
| 3 | 3 / 13 (23.1%) |
| Tumour Regression Grade (CAP) |  |
| 1 | 3 / 12 (25.0%) |
| 2 | 3 / 12 (25.0%) |
| 3 | 7 / 12 (58.3%) |
| MD Anderson Regression Grade (MDA) |  |
| 1 | 3 (23.1%) |
| 2 | 10 (76.9%) |
| Tumour Budding (ITBCC) |  |
| BD1 | 8 / 13 (61.5%) |
| BD2 | 3 / 13 (23.1%) |
| BD3 | 2 / 13 (15.4%) |
| Total Lymph Node (LN) yield | 27.15 (11.80) |
| Positive LN |  |
| 0 | 6 / 13 (46.2%) |
| 1 | 1 / 13 (7.7%) |
| 2 | 2 / 13 (15.4%) |
| 3 | 1 / 13 (7.7%) |
| 4 | 2 / 13 (15.4%) |
| 8 | 1 / 13 (7.7%) |
| Presence of Extracapsular Extension | 3 / 13 (23.1%) |
| Neoadjuvant Therapy |  |
| Agent unknown | 5 / 13 (38.5%) |
| FOLFIRINOX | 8 / 13 (61.5%) |
| ^1^Mean (SD); n / N (%) | |

**Table S2.** Expression of TGF- and BMP-signalling molecules in Tumour Centre (TC) and Front (TF) in tissue of patients who received neoadjuvant treatment (n=11 analysed).

| **Region** | **PDAC_ID1** | **Stroma_ID1** | **PDAC_pSMAD2** | **Stroma_pSMAD2** |
| --- | --- | --- | --- | --- |
| TC | 70.8% (33-88.7, SD = 18.7%) | 24.7% (6.7-56.7, SD = 19.3%) | 21.2% (0-81.8, SD = 26.8%) | 19.4% (0.6-65.6, SD = 22.4%) |
| TF | 64.1% (16.1-100, SD = 27.5%) | 24.7% (9.8-40.2, SD = 11.5%) | 29.2% (0-81.2, SD = 33.9%) | 38.4% (1.2-87.3, SD = 35.9%) |

| **Region** | **PDAC_GREM1** | **Stroma_GREM1** | **PDAC_TGFA** | **Stroma_TGFA** | **PDAC_TGFB1** | **Stroma_TGFB1** | **PDAC_TGFB2** | **Stroma_TGFB2** | **PDAC_BMP4** | **Stroma_BMP4** |
| --- | --- | --- | --- | --- | --- | --- | --- | --- | --- | --- |
| TC | 1.2 (0-6, SD = 2.2) | 1.1 (0-4.8, SD = 1.8) | 1.7 (0.3-7.8, SD = 2.3) | 1.3 (0.2-3.8, SD = 1.1) | 2.4 (0.5-8.1, SD = 2.9) | 1 (0.2-2.6, SD = 0.8) | 1.8 (0.2-6.7, SD = 2.7) | 1.6 (0.5-5.1, SD = 1.5) | 0.6 (0.1-3.2, SD = 1) | 0.6 (0-2.4, SD = 0.7) |
| TF | 0.1 (0-0.7, SD = 0.2) | 0.2 (0-0.8, SD = 0.3) | 1 (0.1-2.1, SD = 0.6) | 1.4 (0.4-2.8, SD = 0.8) | 1.5 (0.6-2.2, SD = 0.5) | 0.7 (0.1-1.5, SD = 0.4) | 0.6 (0.2-0.9, SD = 0.2) | 1 (0.3-1.8, SD = 0.5) | 0.3 (0-0.9, SD = 0.3) | 0.2 (0-0.5, SD = 0.2) |

**Table S3.** Spearman-Rho (ρ) and p-values (P) of correlation analyses. Signalling molecule expression intra- and cross-compartment of interest (neoadjuvant cases excluded).

Correlation coefficients (Cor) and p-values (P) for expression in and **among PDAC parenchyma**.

|  | **Cor_ID1** | **Cor_TGFA** | **Cor_TGFB1** | **Cor_TGFB2** | **Cor_GREM1** | **Cor_BMP4** | **Cor_pSMAD2** | **P_ID1** | **P_TGFA** | **P_TGFB1** | **P_TGFB2** | **P_GREM1** | **P_BMP4** | **P_pSMAD2** |
| --- | --- | --- | --- | --- | --- | --- | --- | --- | --- | --- | --- | --- | --- | --- |
| ID1 | 1.000 | 0.088 | -0.043 | 0.064 | -0.146 | 0.125 | 0.013 |  | 0.183 | 0.514 | 0.334 | **0.028** | 0.059 | 0.849 |
| TGFA | 0.088 | 1.000 | 0.511 | 0.320 | 0.130 | 0.474 | 0.026 | 0.183 |  | **0.000** | **0.000** | 0.052 | **0.000** | 0.699 |
| TGFB1 | -0.043 | 0.511 | 1.000 | 0.301 | 0.345 | 0.174 | 0.145 | 0.514 | **0.000** |  | **0.000** | 0.000 | **0.008** | **0.029** |
| TGFB2 | 0.064 | 0.320 | 0.301 | 1.000 | 0.149 | 0.177 | -0.074 | 0.334 | **0.000** | **0.000** |  | 0.026 | **0.007** | 0.266 |
| GREM1 | -0.146 | 0.130 | 0.345 | 0.149 | 1.000 | 0.091 | 0.111 | **0.028** | 0.052 | **0.000** | 0.026 |  | 0.173 | 0.098 |
| BMP4 | 0.125 | 0.474 | 0.174 | 0.177 | 0.091 | 1.000 | 0.112 | 0.059 | **0.000** | **0.008** | **0.007** | 0.173 |  | 0.091 |
| pSMAD2 | 0.013 | 0.026 | 0.145 | -0.074 | 0.111 | 0.112 | 1.000 | 0.849 | 0.699 | **0.029** | 0.266 | 0.098 | 0.091 |  |

Correlation coefficients (Cor) and p-values (P) for expression **in and among PDAC-associated stroma**.

| **Row** | **Cor_ID1** | **Cor_TGFA** | **Cor_TGFB1** | **Cor_TGFB2** | **Cor_GREM1** | **Cor_BMP4** | **Cor_pSMAD2** | **P_ID1** | **P_TGFA** | **P_TGFB1** | **P_TGFB2** | **P_GREM1** | **P_BMP4** | **P_pSMAD2** |
| --- | --- | --- | --- | --- | --- | --- | --- | --- | --- | --- | --- | --- | --- | --- |
| ID1 | 1.000 | 0.074 | 0.019 | 0.102 | 0.015 | -0.108 | 0.228 |  | 0.216 | 0.750 | 0.089 | 0.810 | 0.072 | 0.000 |
| TGFA | 0.074 | 1.000 | 0.565 | 0.495 | 0.127 | 0.427 | 0.040 | 0.216 |  | 0.000 | 0.000 | 0.036 | 0.000 | 0.509 |
| TGFB1 | 0.019 | 0.565 | 1.000 | 0.510 | 0.252 | 0.421 | -0.054 | 0.750 | 0.000 |  | 0.000 | 0.000 | 0.000 | 0.375 |
| TGFB2 | 0.102 | 0.495 | 0.510 | 1.000 | -0.092 | 0.299 | -0.042 | 0.089 | 0.000 | 0.000 |  | 0.128 | 0.000 | 0.484 |
| GREM1 | 0.015 | 0.127 | 0.252 | -0.092 | 1.000 | 0.132 | 0.131 | 0.810 | 0.036 | 0.000 | 0.128 |  | 0.031 | 0.031 |
| BMP4 | -0.108 | 0.427 | 0.421 | 0.299 | 0.132 | 1.000 | -0.120 | 0.072 | 0.000 | 0.000 | 0.000 | 0.031 |  | 0.045 |
| pSMAD2 | 0.228 | 0.040 | -0.054 | -0.042 | 0.131 | -0.120 | 1.000 | 0.000 | 0.509 | 0.375 | 0.484 | 0.031 | 0.045 |  |

Correlation coefficients (Spearman’s ρ) and p-values for expression **among PDAC-parenchyma and associated stroma**.

| **PDAC** | **Stroma** | **ρ** | **p-value** |
| --- | --- | --- | --- |
| ID1 PDAC | ID1 Stroma | 0.325 | ****0**** |
| ID1 PDAC | TGFA Stroma | -0.005 | 0.938 |
| ID1 PDAC | TGFB1 Stroma | 0.121 | 0.068 |
| ID1 PDAC | TGFB2 Stroma | 0.060 | 0.368 |
| ID1 PDAC | GREM1 Stroma | -0.003 | 0.968 |
| ID1 PDAC | BMP4 Stroma | -0.038 | 0.565 |
| ID1 PDAC | pSMAD2 Stroma | 0.031 | 0.641 |
| TGFA PDAC | ID1 Stroma | 0.074 | 0.262 |
| TGFA PDAC | TGFA Stroma | 0.642 | ****0**** |
| TGFA PDAC | TGFB1 Stroma | 0.358 | ****0**** |
| TGFA PDAC | TGFB2 Stroma | 0.114 | 0.085 |
| TGFA PDAC | GREM1 Stroma | 0.123 | 0.066 |
| TGFA PDAC | BMP4 Stroma | 0.238 | ****0**** |
| TGFA PDAC | pSMAD2 Stroma | 0.008 | 0.904 |
| TGFB1 PDAC | ID1 Stroma | 0.006 | 0.931 |
| TGFB1 PDAC | TGFA Stroma | 0.560 | ****0**** |
| TGFB1 PDAC | TGFB1 Stroma | 0.572 | ****0**** |
| TGFB1 PDAC | TGFB2 Stroma | 0.296 | ****0**** |
| TGFB1 PDAC | GREM1 Stroma | 0.184 | ****0.006**** |
| TGFB1 PDAC | BMP4 Stroma | 0.313 | ****0**** |
| TGFB1 PDAC | pSMAD2 Stroma | 0.110 | 0.099 |
| TGFB2 PDAC | ID1 Stroma | 0.269 | ****0**** |
| TGFB2 PDAC | TGFA Stroma | 0.282 | ****0**** |
| TGFB2 PDAC | TGFB1 Stroma | 0.182 | ****0.006**** |
| TGFB2 PDAC | TGFB2 Stroma | 0.638 | ****0**** |
| TGFB2 PDAC | GREM1 Stroma | -0.051 | 0.443 |
| TGFB2 PDAC | BMP4 Stroma | 0.201 | ****0.002**** |
| TGFB2 PDAC | pSMAD2 Stroma | -0.010 | 0.885 |
| GREM1 PDAC | ID1 Stroma | -0.118 | 0.079 |
| GREM1 PDAC | TGFA Stroma | 0.398 | ****0**** |
| GREM1 PDAC | TGFB1 Stroma | 0.424 | ****0**** |
| GREM1 PDAC | TGFB2 Stroma | 0.037 | 0.581 |
| GREM1 PDAC | GREM1 Stroma | 0.675 | ****0**** |
| GREM1 PDAC | BMP4 Stroma | 0.261 | ****0**** |
| GREM1 PDAC | pSMAD2 Stroma | 0.140 | ****0.037**** |
| BMP4 PDAC | ID1 Stroma | 0.090 | 0.177 |
| BMP4 PDAC | TGFA Stroma | 0.419 | ****0**** |
| BMP4 PDAC | TGFB1 Stroma | 0.153 | ****0.021**** |
| BMP4 PDAC | TGFB2 Stroma | 0.289 | ****0**** |
| BMP4 PDAC | GREM1 Stroma | 0.042 | 0.532 |
| BMP4 PDAC | BMP4 Stroma | 0.604 | ****0**** |
| BMP4 PDAC | pSMAD2 Stroma | 0.012 | 0.858 |
| pSMAD2 PDAC | ID1 Stroma | -0.061 | 0.358 |
| pSMAD2 PDAC | TGFA Stroma | 0.037 | 0.573 |
| pSMAD2 PDAC | TGFB1 Stroma | 0.196 | ****0.003**** |
| pSMAD2 PDAC | TGFB2 Stroma | -0.155 | ****0.018**** |
| pSMAD2 PDAC | GREM1 Stroma | 0.261 | ****0**** |
| pSMAD2 PDAC | BMP4 Stroma | -0.043 | 0.52 |
| pSMAD2 PDAC | pSMAD2 Stroma | 0.471 | ****0**** |

**Table S4.** Minimal to maximal count (mean/median) of tumour buds per tissue core for region and target investigated (non-neoadjuvant patients only).

|  | **GREM1** | **BMP4** | **ID1** | **pSMAD2** | **TGF-A** | **TGF-B1** | **TGF-B2** |
| --- | --- | --- | --- | --- | --- | --- | --- |
| **Tumour Centre** | 0.0 - 14.0 (1.9 / 0.0) | 0.0 - 12.0 (1.8 / 1.0) | 0.0 - **24.0** (**2.0** / 0.0) | 0.0 - 12.0 (1.3 / 0.0) | 0.0 - 15.0 (1.0 / 0.0) | 0.0 - 15.0 (1.2 / 0.0) | 0.0 - 16.0 (1.1 / 0.0) |
| **Tumour Front** | 0.0 - 13.0 (1.7 / 0.0) | 0.0 - 11.0 (1.7 / 1.0) | 0.0 - 15.0 (1.4 / 0.0) | 0.0 - 13.0 (0.9 / 0.0) | 0.0 - 7.0 (0.7 / 0.0) | 0.0 - 11.0 (1.1 / 0.0) | 0.0 - 14.0 (1.0 / 0.0) |
| **Stroma** | 0.0 - 20.0 (1.3 / 0.0) | 0.0 - 11.0 (1.6 / 0.0) | 0.0 - 17.0 (1.7 / 0.0) | 0.0 - 16.0 (1.4 / 0.0) | 0.0 - 13.0 (1.1 / 0.0) | 0.0 - 11.0 (0.6 / 0.0) | 0.0 - 12.0 (0.8 / 0.0) |

**Table S5.** Binary quantification of immune infiltrate (absolute numbers; see also **Figure S5**) and “heterogeneity” (e.g. TC: CD68^high^ but TF: CD68^low^, concordant would be CD68^high^ in both TC and TF) among tumour regions (non-neoadjuvant patients only).

|  | **Tumour Centre (TC)** | | **Tumour Front (TF)** | |
| --- | --- | --- | --- | --- |
|  | Low | High | Low | High |
| CD68 | 59 (78.7%) | 16 (21.3%) | 51 (65.4%) | 27 (34.6%) |
| CD163 | 68 (75.6%) | 22 (24.4%) | 67 (70.5%) | 28 (29.5%) |
| CD8 | 68 (79.1%) | 18 (20.9%) | 56 (61.5%) | 35 (38.5%) |
| FOXP3 | 73 (83.9%) | 14 (16.1%) | 79 (83.2%) | 16 (16.8%) |

|  | **Immune heterogeneous cases (%)** | **% concordant low/high in TC/TF** |
| --- | --- | --- |
| CD68 | 21 (20.19%) | 79.81 |
| CD163 | 29 (27.88%) | 72.12 |
| CD8 | 27 (25.96%) | 74.04 |
| FOXP3 | 16 (15.38%) | 84.62 |

**Table S6.** Other univariate survival analysis (neoadjuvant patients excluded). P-values when stratified by mean transcript numbers/expression percentage (log-rank test, p<0.05 in bold). *TC*: Tumour Centre, *TF*: Tumour Front.

|  | **Stroma** | | **PDAC** | |
| --- | --- | --- | --- | --- |
|  | TC | TF | TC | TF |
| **BMP4** | p=0.17 | p=0.88 | p=0.27 | p=0.19 |
| **GREM1** | p=0.66 | p=0.77 | p=0.7 | p=0.52 |
| **ID1** | p=0.99 | p=0.37 | p=0.21 | p=0.26 |
| **pSMAD2** | p=0.32 | p=0.62 | p=0.67 | p=0.7 |
| **TGF-A** | p=0.46 | p=0.069 | p=0.58 | p=0.86 |
| **TGF-B1** | p=0.35 | p=0.49 | p=0.99 | p=0.16 |
| **TGF-B2** | **p=0.019** | p=0.88 | p=0.27 | p=0.19 |

**Table S7.** Performance metrics of segmentation model, including both aggregate metrics (Precision, Recall, F1-Score) and confusion matrix components (True Positives, False Positives, False Negatives, True Negatives) for each class.

|  | **Precision** | **Recall** | **F1-Score** | **True Positives** | **False Positives** | **False Negatives** | **True Negatives** |
| --- | --- | --- | --- | --- | --- | --- | --- |
| **stroma** | 0.9920 | 0.6269 | 0.7683 | 3,854,081 | 31,049 | 2,293,509 | 2,455,748 |
| **epithelium** | 0.9441 | 0.8949 | 0.9189 | 2,197,710 | 130,081 | 258,038 | 6,147,590 |
| **Image (overall)** | 0.9681 | 0.7609 | 0.8436 | 3,025,895 | 1,275,773 | 1,275,773 | 81,731,711 |
